# A dual role of dLsd1 in oogenesis: regulating developmental genes and repressing transposons

**DOI:** 10.1093/nar/gkz1142

**Published:** 2019-12-04

**Authors:** Julie M J Lepesant, Carole Iampietro, Eugenia Galeota, Benoit Augé, Marion Aguirrenbengoa, Clemèntine Mercé, Camille Chaubet, Vincent Rocher, Marc Haenlin, Lucas Waltzer, Mattia Pelizzola, Luisa Di Stefano

**Affiliations:** 1 LBCMCP, Centre de Biologie Intégrative (CBI), Université de Toulouse, CNRS, UPS, Toulouse 31062, France; 2 Center for Genomic Science of IIT@SEMM, Fondazione Istituto Italiano di Tecnologia (IIT), Milan 20139, Italy; 3 CBD, Centre de Biologie Intégrative (CBI), Université de Toulouse, CNRS, UPS, Toulouse 31062, France; 4 School of Biological Sciences, University of Western Australia, Perth, WA 6009, Australia; 5 Université Clermont Auvergne, CNRS, INSERM, GReD, Clermont-Ferrand F-63000, France

## Abstract

The histone demethylase LSD1 is a key chromatin regulator that is often deregulated in cancer. Its ortholog, dLsd1 plays a crucial role in Drosophila oogenesis; however, our knowledge of dLsd1 function is insufficient to explain its role in the ovary. Here, we have performed genome-wide analysis of dLsd1 binding in the ovary, and we document that dLsd1 is preferentially associated to the transcription start site of developmental genes. We uncovered an unanticipated interplay between dLsd1 and the GATA transcription factor Serpent and we report an unexpected role for Serpent in oogenesis. Besides, our transcriptomic data show that reducing dLsd1 levels results in ectopic transposable elements (TE) expression correlated with changes in H3K4me2 and H3K9me2 at TE loci. In addition, our results suggest that dLsd1 is required for Piwi dependent TE silencing. Hence, we propose that dLsd1 plays crucial roles in establishing specific gene expression programs and in repressing transposons during oogenesis.

## INTRODUCTION

Histone methylation plays a key role in the regulation of transcription and in the formation of heterochromatin. Dynamic regulation of histone methylation by the activity of histone methyltransferases and demethylases confers plasticity to chromatin-related processes. The histone lysine demethylase 1 (LSD1) has emerged as a key chromatin regulator essential for normal development and implicated in cancer.

LSD1, also known as KDM1, was the first histone demethylase to be discovered (1). LSD1 functions as a transcriptional co-repressor as part of the coREST and NuRD complexes by removing the active H3K4 mono and dimethyl marks from promoters and enhancers (1–4). However, LSD1 has also been reported to function as a co-activator of nuclear hormone receptors by mediating demethylation of the repressive H3K9 methyl mark (5). LSD1 dual substrate specificity has been proposed to determine its activity as a repressor or activator of transcription and it has been ascribed to interaction with specific co-factors, chromatin context (6) and, more recently, to LSD1 alternative splicing (7,8).

LSD1 is essential for mouse viability (9) and is required for pituitary, hematopoietic (10,11) and osteogenic (12) differentiation. In embryonic stem cells (ESC), LSD1 promotes the silencing of the ESC gene expression program and its depletion impairs differentiation (4).

In *Drosophila*, dLsd1 loss-of-function leads to reduced viability, wing and bristle defects as well as complete sterility associated with ovarian defects (13). The rudimentary ovaries of *dLsd1* mutant females have an abnormal number of germ-line stem cells and follicle cells (13–15) indicating that dLsd1 plays essential roles in oogenesis. However, the precise mechanisms by which dLsd1 controls different aspects of oogenesis still needs to be elucidated.

Previous ChIP-Seq studies using an ectopically expressed and tagged form of dLsd1 suggest that dLsd1 controls the number of germ line stem cells by regulating the expression of a specific set of genes in Escort Cells (ECs) and cap cells, two specialized set of somatic cells present in the anterior part of the Drosophila ovary germarium (16). However, use of an ectopically expressed and tagged form of dLsd1 could alter target specificity and endogenous dLsd1 might compete with the ectopically expressed form resulting in loss of information. In addition, dLsd1 expression in the ovary is ubiquitous and thus is not limited to these two cell populations (14). Consistently, dLsd1 was shown to affect epigenetic plasticity in late follicle progenitor in the ovary by controlling H3K4me levels (15) but its precise mechanism of action remains unknown. Determining the full set of genes regulated by dLsd1 in ovary is instrumental to understanding its role in oogenesis.

Here, we profiled dLsd1’s binding sites on chromatin by ChIP-Seq using an antibody that recognizes endogenous dLsd1. Moreover, we characterized changes in the transcriptional landscape of ovaries depleted of dLsd1 compared to their wild-type counterpart genome-wide. We find that dLsd1 is preferentially bound to the TSS of multiple genes with known developmental roles and that more than one third of dLsd1 peaks contains a CGATA motif. This motif is recognized by a family of transcription factors with key regulatory function in development, the GATA family (17). Accordingly, we were able to show that a member of the GATA family, Serpent (Srp) contributes (directly or indirectly) to dLsd1 recruitment to a subset of GATA motif containing genes. This led us to discover a novel role for Srp in oogenesis.

One final, exciting aspect of our study is the discovery that dLsd1 depletion results in de-repression of transposable elements through changes of their chromatin state. Interestingly, our genetic analyses indicate that dLsd1 is required for Piwi dependent TE repression. Silencing of transposons is critical for oogenesis and their aberrant expression has been implicated in sterility (18). In light of our results, we suggest that dLsd1 plays multiple roles during oogenesis including the regulation of key developmental genes, among which Serpent's targets, and the silencing of transposable elements.

## MATERIALS AND METHODS

### Drosophila strains

The *Actin5C-GAL4, tub-GAL80*^ts^, *Twist-GAL4, UAS-RNAi Luciferase* and the *w^1118^*stocks were obtained from the Bloomington Stock center *(BL#4414, BL#7018, BL#31603, BL#5905)*. The *Traffic-Jam-GAL4* line and the *Nos-GAL4>UAS-Dicer* line were gifts of Nicola Iovino. The *UAS-Tomato-Piwi* line was a gift of Chantal Vaury (19). Transgenic *UAS-RNAi* lines, *gypsy-lacZ* reporters and the GFP tagged *serpent* transgenic line were obtained from the Vienna Drosophila Research Center (VDRC) (accession numbers: 25218/GD; 35578/GD; 109521/KK, 313222, 313223, 318053*). dLsd1^Δ^^N^* flies are described in (13)*. w^1118^* flies were used as wild-type control in all experiments. Flies were grown on standard *Drosophila* medium and maintained at 25°C unless specified otherwise.

### Temperature shift experiments

Crosses were established and cultured at 25°C, the permissive temperature, until late pupal stages. Progenies were divided into two equal pools; controls were cultured at 25°C and the experimental group was shifted to 30°C for 4 days. Cultures were transferred onto fresh food augmented with yeast paste for 2 days prior to ovary dissection.

### Egg laying assay

Female and male adult flies were collected within 24 h of eclosion and transferred to an agar plate with wet yeast paste to prime females for egg laying. Flies were incubated at 25°C for 24 h and transferred to a new agar plate. The number of eggs on the plate was counted on days 2 and 3. At least 10 females flies were individually tested for each genotype.

### Cell lines

S2 and Kc167 cells were grown in Schneider's Drosophila medium supplemented with 10% fetal calf serum (FCS) and penicillin/streptomycin. OSS cells were grown in M3 medium supplemented with 10% fetal calf serum (FCS), 0.6 mg/ml glutathione (SIGMA), 10 milliunits/ml insulin (SIGMA) and fly extract (DGRC).

### RNA extraction and Microarray

Total RNA was extracted using Trizol (Invitrogen) from three independent preparations of ovaries dissected from 3 to 6 days old *Drosophila* females maintained for 2 days on yeast. RNA was treated with DNA-free™ DNA Removal Kit (Ambion) as described by the manufacturer. RNA concentration and purity were assessed using a NanoDrop spectrophotometer and RNA integrity was verified with a Bioanalyzer 2100 (Agilent). Gene expression analysis was performed by the GenoToul platform in Toulouse. Briefly, biotin-labeled cRNA was synthesized using the Low Input Quick Amp Labeling kit (Agilent). Agilent Drosophila Gene Expression Microarrays (4 × 44K) were hybridized with 15 μg of labeled cRNAs. The slides (Drosophila Gene Expression Microarray, 4 × 44K ref 021791) were hybridized following the One-Color Microarray-Based Gene Expression Analysis Protocol (Agilent) and scanned on a Tecan MS200 scanner. The median signal of each spot in the hybridized arrays was determined and quantified using Feature Extraction software v11.5.1.1. The data from all the microarrays were transformed in log_2_ intensity. Then they were normalized together using the ‘limma’ package. Statistical analyses were performed using the R software package.

### Chromatin immunoprecipitation coupled with deep sequencing (ChIP-Seq)

ChIP assay was performed in triplicate as described previously (20) using the anti-dLsd1 antibody (13) and the corresponding pre-immune serum (13). Approximately 1000 ovary pairs were used in each ChIP-Seq experiment from 3 to 6 days old *w^1118^* females raised at 25°C on standard Drosophila medium. The quality of DNA precipitate was checked with the Bioanalyzer and by real-time qPCR before sequencing. Input samples for each genotype were prepared and sequenced in parallel with DNA precipitated with the anti-dLsd1 antibody and the pre-immune serum. For the libraries preparation, ChIP DNA (10 ng) was blunt-ended and phosphorylated, and a single ‘A’ nucleotide was added to the 3′ ends of the fragments in preparation for ligation to an adapter that has a single-base ‘T’ overhang. The ligation products were purified and size-selected by Agencourt AMPure XP beads. Purified DNA was PCR-amplified to enrich for fragments that have adapters on both ends. All these steps were performed on an automation instrument, Biomek FX by Beckman Coulter. The final purified products were verified prior to cluster generation on a Bioanalyzer 2100. Libraries were sequenced in the single read mode on a HiSeq 2000 sequencer (Illumina).

### Bioinformatic analysis

The HTS-flow management system was used for the processing and alignment of the sequencing reads (21). Reads alignment was performed to the dm6 reference genome (https://www.ncbi.nlm.nih.gov/assembly/GCF_000001215.4/, BDGP6 build based on the Flybase release 6.13) using BWA with default parameters (21,22). After the alignment, duplicate reads, having identical start and end genomic coordinates, were removed using samtools. Two lists of dLsd1 significantly enriched peaks were obtained with MACS2 (23) corresponding to the use of two different backgrounds (input and pre-immune), choosing the nomodel option and setting a q-value cutoff of 1e-02.

For each one of the two lists (dLsd1 vs input) and (dLsd1 vs pre-immune), consensus-peaks from the three replicates were obtained using the intersect function of the GenomicRanges Bioconductor package. The peaks were individually called for both experiments (dLsd1 versus input and dLsd1 versus pre-immune) using bedtools (v2.25.0). We decided to use the list of peaks normalized against the pre-immune serum for further analysis (Supplementary Table S1), as the pre-immune serum derives from the same animal in which our antibody was raised and it allows us to control for antibody cross-reactivity.

Genomic annotation of the peaks was obtained from the GRannotateSimple function of the CompEpiTools Bioconductor package, which, based on the overlap with promoters or gene bodies classifies peaks as ‘promoters’, ‘intragenic’ or ‘intergenic’. Promoter regions were defined as regions at 1 KB upstream and 500 bp downstream of the TSS of transcripts.

Peaks width was calculated using peaks positions and plotted in R. In order to represent the mean signal per base around the TSS, for the three datasets (input, pre-immune, dLsd1), we calculated the coverage in a windows of [−1 kb; +2 kb] around the TSS position (refGene) corresponding to dLsd1 specific peaks nearest genes.

Genes were classified according to the modENCODE RNASeq analysis in ovaries (Accession: SRX494116, SRX494041, SRX014987) on the basis of their reads per kilobase per million (rpkm). We defined them as highly expressed (rpkm > 100 ; *n* = 574), moderately expressed (100 < rpkm < 10, *n* = 3650), lowly expressed (10 < rpkm < 1, *n* = 2691), not expressed (rpkm < 1, *n* = 9620). The mean coverage per base was then normalized using the input mean signal calculated on the same [−1 kb; +2 kb] window around the TSS.

To determine whether dLsd1 peaks overlap with H3K4me2, H3K9me2 and Pol2 Ser5-P, ChIP-chip data and ChIP-Seq data from ovary samples were downloaded from the GEO website (Accession: H3K4me2: GSE45524, H3K9me2: GSE45522, Pol2 Ser5-P: SRP018251) and analyzed with ComEpiTools or BedTools v2.25.0 (22). Two peaks were considered common between the samples if they had at least 1 base overlapping (bedtools v2.25.0). The coordinates of the euchromatin-heterochromatin boundaries were obtained from Hoskins *et al.* (24).

Gene Ontology analysis was performed using DAVID v6.8 (25). Presented adjusted *P*-values have been calculated using the Bonferroni or Benjamini–Hochberg method.

Motif discovery was performed with significant dLsd1 peaks (peak summit ± 250 bp) using MEME-ChIP (v4.11.3) from the MEME suite with default settings (26,27). Motifmatch R coupled with a fisher test was used to ensure that the motifs are statistically enriched at dLsd1 peaks compared to an equal number of random DNA sequences of the same length.

We have used RepEnrich2 (28) to estimate the enrichment of repetitive elements in our samples. To this end, we downloaded the repetitive element annotation for Drosophila from Repeatmasker. The annotation file contains the genomic coordinates of repetitive elements, their associated identifiers, the class and family of the repeat. During the alignment phase RepEnrich separates each sample's reads in two different files, one for the uniquely mapping and one for the multi-mapping. Uniquely mapping reads are summed with fractional counts of the multi-mapping reads. We used edgeR with the method GLM to compute the enrichment of reads in anti-dLsd1 samples against input or pre-immune. Supplementary Table S9 reports the fold changes with the False Discovery Rates (FRD) of repeats for dLsd1 samples against input and pre-immune.

### ChIP-qPCR

Drosophila S2 cells were fixed in 1% formaldehyde for 15 min at room temperature; the reaction was stopped by addition of glycine, and cells were washed in PBS and harvested in IP buffer (1 volume of SDS buffer to 0.5 volume of Triton dilution buffer and protease inhibitors).

Drosophila ovaries were dissected and fixed in 1 ml of 1% formaldehyde at room temperature for 10 min. The crosslinking was stopped by the addition of the same volume of 1.25M glycine solution. Pellets were resuspended in sonication buffer (1%Triton X-100, 0.1% Deoxycholate, 50 mM Tris 8.1, 150 mM NaCl, 5 mM EDTA). Chromatin was sonicated to an average size of 750 bp. Sonicated samples were then blocked by adding Protein G and A sepharose beads and incubating at 4°C for one hour. The beads were removed. 10% of the sample was kept aside as INPUT and to the remaining sample 10 μl of dLsd1 antibody or pre-immune serum, 5 μl of Serpent antibody or IgG (1μg) was added and incubated overnight at 4°C. The next day protein A and G sepharose beads were added and incubated for 2 h at 4°C. After extensive washes, immunocomplexes were eluted from the beads and cross-links were reversed. The DNA was recovered by phenol-chloroform extraction and ethanol precipitation. DNA was resuspended in 150 μl of water, and 7.5 μl was used for real-time qPCRs.

### Real time quantitative PCR (RT-qPCR)

Real-time PCR analyses were performed in triplicate as described previously (20). Graphs representing RT-qPCR data contain averages and standard deviations. Primer sequences are provided in Supplementary Tables S2 and S3.

### RNAi in Drosophila cells

Double-stranded RNA (dsRNA) for RNAi experiments was generated using a RiboMax large-scale RNA production system (Promega) according to the manufacturer's instructions. 4 × 10^6^ Drosophila S2 or Kc167 cells were incubated with 20 μg of dsRNA for 4 days for dsSrp and dsGatad. In the case of dsLsd1, a second incubation with 20 μg of dsRNA was performed at 4 days and cells were harvested 2 days later. Primer sequences to generate the dsRNA are provided in Supplementary Table S4.

### X-gal staining

Ovaries were dissected in PBS, fixed in 4% paraformaldehyde/PBS at room temperature for 5 min and rinsed three times with PBS-T (0.3% triton). They were then incubated in staining solution (10 mM sodium phosphate buffer pH 7.2, 1 mM MgCl_2_, 150 mM NaCl, 3 mM potassium ferricyanide, 3 mM potassium ferrocyanide, 0.3% Triton, 0.2% X-Gal) at 37°C over night. Images were taken with a Nikon Camera DXM1200c on a Nikon Eclipse 80i microscope.

### Immunoprecipitation

S2 cells and ovaries were collected, washed in PBS and resuspended in E1A Buffer (50 mM HEPES, 250 mM NaCl, 0.1% NP40, 1 mM DTT, supplemented with protease inhibitor cocktail, Roche) or, for samples treated with Benzonase, in modified E1A Buffer (50 mM HEPES, 250 mM NaCl, 0.1% NP40, 2.0 mM MgCl_2_, supplemented with protease inhibitor cocktail, Roche). Samples were sonicated two times for 10 s at amplitude 30 (Bioblock Scientific Ultrasonic processor) and when indicated, samples were incubated overnight in the presence of 1500 units (ovarian extract) or 1000 units (OSS and S2 extract) of Benzonase (SIGMA). DNA digestion was confirmed by running the extract on an agarose gel stained with ethidium bromide. The extracts were cleared by centrifugation at 13 000g for 15 min at 4°C. Between 0.4 mg and 1.5 mg of proteins were pre-adsorbed with 30 μl of sepharose beads slurry (Sigma) for 1 h at 4°C before being incubated with one of the following antibodies: 5 μl of anti-GFP (Chromotek), 10 μl anti-dLsd1 antibody, 10 μl anti-Piwi antibody (Santa-Cruz). Rabbit and mouse IgG were used as controls. The beads were spun down and washed in IP buffer. Immunoprecipitated proteins were processed for SDS-PAGE and western blot analyses (20 μg of proteins for input proteins and the totality of immunoprecipitated proteins) using standard techniques.

### Western blot

Kc167 and ovaries were collected, washed in PBS and resuspended in E1A Buffer (50 mM HEPES, 250 mM NaCl, 0.1% NP40, 1 mM DTT, 0.2 mM PMSF, 1 μg/μl Leupeptin, 1 μg/μl Aprotinin). After a centrifugation, proteins were recovered from supernatant. Immunoblots were performed using standard procedure. The blots were developed by photo-luminescence procedure using Super Signal West Dura 34075 (Thermo Scientific) and Chemidoc Touch Imaging System (BioRad).

### Antibodies

Antibodies used in this study include anti-dLsd1 (13), IgG from rabbit serum (I5006, Sigma), anti-H3 (ab1791, Abcam), anti-H3K4me1 (ab8895, Abcam), anti-H3K4me2 (ab33256, Abcam), anti-H3K4me3 (ab8580, Abcam), anti-H3K9me2 (ab1220, Abcam), anti-H3K27me3 (ab6002, Abcam), anti-tubulin (T6199, Sigma), anti-GFP (Chromotek), anti-Rpd3 (sc-30559, Santa-Cruz), anti-Piwi (sc-390946, Santa-Cruz), anti-HA (12CA5, Sigma). Polyclonal anti-Srp antibody was generated by rabbit immunization with a RKRKPKGTKSEKSK peptide (amino acids 850–863 of Srp-PA; Eurogentec), purified on Protein A Sepharose beads (Sigma-Aldrich) and concentrated on Amicon Ultra-0.5 100 kDa column (Sigma-Aldrich). Validation of the antibody specificity was carried out in Kc167 cells as shown in Supplementary Figure S3D.

### Transfections

*Drosophila* S2 cells were transfected for 72h hours using the FUGENE reagent (PROMEGA) as per manufacturer instructions.

### Plasmids

The following plasmids were used to transfect S2 cells: pAcGFP (gift from F. Roch), and pAcGFP-dLsd1. To obtain the pAcGFP-dLsd1 construct, the dLsd1 cDNA was amplified from the LD45081 clone from the Drosophila Genomic Research Center (DGRC) and cloned into pAcGFP using Age1 and Not1 restriction enzymes from New England Biolabs.

### *In situ* hybridization

Probes for *in situ* hybridization were prepared as described in (29). *In situ* hybridization was performed as described in (30). For Digoxigenin labelled RNA probes synthesis, we used pBS-Srp (gift from C. Antoniewski). Briefly, ovaries were fixed for 30 min with 200 μl of 4% paraformaldehyde (PAF) in PBS, 20 μl of DMSO and 600 μl of heptane. They were washed in PBS Tween 0.1% (PBT), digested 20 min with 0.05 mg/ml proteinase K (in 50 mM Tris–HCl pH 7.5 5 mM EDTA), post-fixed 20 min with 4% PAF in PBS, dehydrated 1 h at −20°C in methanol/DMSO (9/1) and progressively rehydrated in PBT. They were pre-incubated for 1 h at 65°C in HB buffer (50% formamide, 2× SSC, 1 mg/ml Torula RNA, 0.05 mg/ml Heparin, 2% Roche blocking reagent, 0.1% CHAPS, 5 mM EDTA, 0.1% Tween 20) and incubated overnight with anti-sense DIG-labeled RNA probes. The samples were washed in HB for 1 h at 65°C, in HB/PBT (50/50) for 20 min at 65°C and several times in PBT at room temperature. Ovaries were incubated for 30 min in PBT–1% BSA before being incubated with anti-DIG antibody conjugated to alkaline phosphatase (Roche, 1:2000 in PBT–BSA) for 2 h. After several washes in PBT, *in situ* hybridization signals were revealed with FastRed (Roche) in Staining Buffer (100 mM NaCl, 50 mM MgCl_2_, 100 mM Tris–HCl pH 8.2 an 0.1% Tween 20).

### Immunostaining

Immunostainings were performed as described in (30). Briefly, ovaries were fixed for 30 min with 4% paraformaldehyde (PAF) in PBS and washed in PBS Triton 0.3% (PBTr). They were permeabilized in PBS 1% Triton for 2 h and blocked in PBTr with 1% BSA for 30 min. They were incubated with primary antibodies at 4°C over-night in PBTr-BSA, washed in PBTr, incubated for 2 h at room temperature with corresponding Alexa Fluor-labeled secondary antibodies and Phalloidin-SR101 (Interchim), washed in PBTr and mounted in Vectashield medium (Eurobio-Vector) following incubation with DAPI. The following antibodies were used: anti-GFP (1:2000), anti-Rabbit coupled to Alexa 488 (1:800). Images were acquired with a ZEISS LSM 710 confocal microscope.

### Statistical analysis

Data are presented as mean ±SEM from at least three independent biological replicates. All statistical analyses between two groups were done by two-sided Student's t test when data followed a normal distribution; otherwise a two-sided Wilcoxon–Mann–Whitney test was applied. Normality was examined using Shapiro test. Statistical analyses were done using R (http://www.R-project.org/).

## RESULTS

### dLsd1 primarily binds TSS

**Figure 1.**
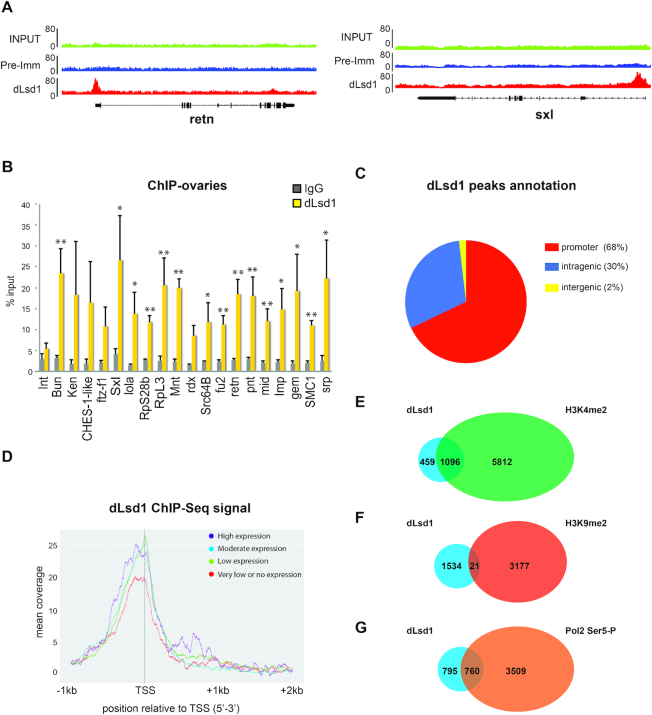
dLsd1 binding is enriched at TSS. (**A**) Integrated Genome Browser (IGB) screenshots of two dLsd1 enriched sites. Anti-dLsd1, pre-immune serum IP and input are shown. (**B**) ChIP-qPCR analysis of dLsd1 binding at a subset of genes identified as bound by dLsd1 in the ChIP-Seq dataset in wild-type (*w^1118^*) ovaries. IgG were used as a control for specificity. Int is an intergenic region used as negative control, and Bun is a previously identified dLsd1 target used as a positive control. The y axis represents enrichment as percent input. The ChIP experiments were performed in triplicate, and error bars indicate standard errors of the means. A Welch two-sample *t*-test was performed to indicate significance (*P < 0.05; **P < 0.01). (**C**) Genomic location of dLsd1 ChIP-Seq peaks. The promoter regions are defined as the sequences from 1KB upstream to 500bp downstream of the transcription start site (TSS). (**D**) dLsd1 ChIP-Seq read density around the TSS of genes classified as highly expressed, moderately expressed, lowly expressed and inactive (very low or no expression) according to the modENCODE RNA-Seq dataset in the ovary. Expression levels were estimated as reads per kilobase per million, rpkm. (**E–G**) Venn diagrams showing the number of common peaks between dLsd1 ChIP-Seq and H3K4me2 ChIP on chip (**E**), H3K9me2 ChIP on chip (**F**) and Polymerase 2 phosphorylated Serine 5 (Pol2 Ser5-P) ChIP-Seq (**G**) peaks in the ovary.

dLsd1 is essential for fertility and its depletion causes strong defects in Drosophila oogenesis (13). To tackle the functions of dLsd1 in oogenesis, we sought to test if it might target specific genes to control their expression. To this end, we performed chromatin immuno-precipitation followed by massively parallel DNA sequencing (ChIP-Seq) in the ovary using a previously validated anti-dLsd1 antibody (13). The analysis of dLsd1 ChIP-Seq data led to the identification of 1555 dLsd1 peaks above the background when normalized against the pre-immune serum IP (Supplementary Table S1). Representative dLsd1 peaks are shown in Figure 1A and Supplementary Figure S1.

To confirm the ChIP-Seq data, we selected a panel of genes bound by dLsd1 according to our ChIP-Seq experiments and performed independent ChIP-qPCR. As shown in Figure 1B, we were able to confirm dLsd1 binding for 14 of the 18 candidate genes tested and for the previously characterized dLsd1 target *Bun* (16) (Figure 1B). An intergenic region (*Int*) was used as negative control, and as expected was not bound by dLsd1 (Figure 1B). Moreover, IgG showed no significant enrichment over any of the regions tested (Figure 1B), supporting the relevance of our ChIP data. Having validated the ChIP-Seq data, we used them to gain further information on dLsd1 binding patterns in the ovary. We first examined the breadth of dLsd1 peaks to determine if dLsd1 is recruited to discrete sites or if binding spreads along the chromatin. The median size of the dLsd1 peak is 335 bp (Supplementary Figure S2A) indicating that dLsd1 is mainly recruited to discrete sites.

To determine where dLsd1 is bound in the genome, we analyzed the overlap of dLsd1 peaks with annotated regions. 68% of dLsd1 peaks were located at promoter regions of annotated genes (defined as the regions between 1Kb upstream and 500 bp downstream of the TSS) (Figure 1C). Importantly, analysis of the distribution of the reads across the promoter shows that dLsd1 binds at or in close proximity to the TSS (Supplementary Figure S2B). We then performed a classification of dLsd1 target genes into highly, moderately, lowly and not expressed on the basis of their expression levels in the ovary according to the modENCODE datasets. These analyses suggest that dLsd1 is bound with a slightly higher affinity to expressed genes (Pearson's Chi-squared test *P* value < 2.2e–16) (Figure 1D). We next performed gene ontology (GO) analysis to assess the functions of the genes bound by dLsd1. GO analysis suggests that dLsd1 binds to a wide variety of genes important for oogenesis, neurogenesis and transcription (Supplementary Figure S2C).

To study the relationship between dLsd1 occupancy and the chromatin environment, we compared dLsd1 peaks to available maps of H3K4me2, H3K9me2 and Pol2 phosphorylated Ser5 distribution in the ovary. We found that the majority of dLsd1 peaks (70%) resides within H3K4me2 domains and that only a small subset of dLsd1 peaks overlaps with H3K9me2 (Figure 1E, F). As expected, the majority of common peaks between dLsd1 and H3K4me2 are located in euchromatic regions (1093/1096). In addition, approximately half of dLsd1 peaks overlap with Pol2 phosphorylated Ser5 (Figure 1G) almost exclusively at euchromatic loci (757/760). These results are consistent with dLsd1 localization around the TSS of expressed genes and raise the possibility that dLsd1 might play a role in the transcriptional regulation of the bound genes.

### GATA motifs are enriched at dLsd1 peaks

**Figure 2.**
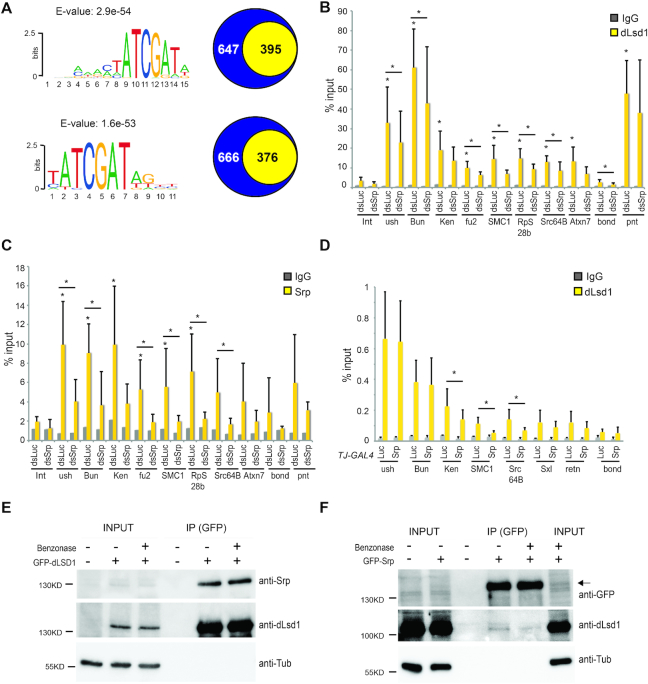
The GATA factor binding motif is enriched at dLsd1 ChIP-Seq peaks. (**A**) dLSD1 peaks MEME analysis. Shown are the sequence logos of the top 2 motifs found by performing an analysis of the sequences of dLsd1 peaks proximal to TSS using the MEME suite program. The E-values corresponding to each motif are indicated. The numbers in the yellow and blue circles of the Venn diagrams indicate respectively how many dLsd1 peaks carry or not the motifs shown. (**B**) Serpent depletion affects dLsd1 binding at a subset of GATA motif carrying genes in S2 cells. ChIP-qPCR analysis of dLsd1 binding at a subset of dLsd1 targets carrying a putative GATA motif in S2 cells treated with either dsRNA against *luciferase* (control) or against *srp*. A Wilcoxon test was performed to indicate significance (*P < 0.05). (**C**) Srp binds a subset of dLsd1 target genes. ChIP-qPCR analysis of Srp binding at a subset of dLsd1 targets carrying a putative GATA motif in S2 cells. IgG were used as a control for specificity. Int is an intergenic region used as negative control, *ush* is a previously identified Serpent target used as a positive control, *pnt* is a dLsd1 target that does not carry a GATA motif and *bond* is a gene which is not supposed to be bound by neither Srp nor dLsd1. All ChIP experiments were performed at least in triplicate, and error bars indicate standard errors of the means. A Wilcoxon test was performed to indicate significance (*P < 0.05). (**D**) Serpent depletion affects dLsd1 binding at a subset of GATA motif carrying genes in ovaries. ChIP-qPCR analysis of dLsd1 binding at a subset of dLsd1 targets carrying a putative GATA motif in ovaries expressing an RNAi against luciferase or srp under the control of the *Traffic Jam GAL4* (*TJGAL4)* driver. *Retn* is a dLsd1 target that does not carry a GATA motif. A Wilcoxon test was performed to indicate significance (*P < 0.05). (**E**) dLsd1 and Srp physically interact. Immunoblot showing co-immunoprecipitation of endogenous Srp with GFP-tagged dLsd1 in S2 cells. The immunoprecipitated GFP-dLsd1 is also shown and Tubulin was used as negative control. Benzonase was added where indicated. (**F**) Immunoblot showing co-immunoprecipitation of endogenous dLsd1 with GFP-tagged Srp in S2 cells. GFP-Srp is also shown (indicated by an arrow). Tubulin was used as a negative control. Benzonase was added where indicated.

Human LSD1 does not bind DNA directly (31–33); rather, it is tethered to DNA through binding to specific co-factors, including CoREST. However, CoREST binding to DNA is weak and unspecific and transcription factors are needed to strengthen the association and provide specificity (34). In *Drosophila*, very little is known about the transcription factors that influence dLsd1 recruitment to specific sets of genes. With the goal of identifying potential new partners of dLsd1, we used MEME suite program and motifmatchR to find transcription factors binding motifs or de novo motifs that are significantly enriched at dLsd1 peaks proximal to the TSS. We obtained a list of 13 enriched motifs (Supplementary Table S5). The top 2 motifs enriched at dLsd1 ChIP-Seq peaks are GATA motifs (Figure 2A), recognized by the GATA family of transcription factors.

The GATA family of transcription factors has been shown to control the development of hematopoietic, neural and cardiac tissues in multiple organisms including Drosophila (35). However, the role of GATA factors in oogenesis is largely unknown. One study showed that dGATAb (Serpent/Srp) is expressed in the ovary (36). To test whether the other GATA factors, Pannier (dGATAa), Grain (dGATAc), dGATAd and dGATAe are also expressed in the ovary, we analyzed the Fly atlas and modENCODE expression data. This analysis indicated that only Srp and dGATAd mRNAs are transcribed in the ovary (Supplementary Table S6). Therefore, we decided to focus on these two factors.

### dLsd1 binding to a set of Serpent motif-containing genes is Serpent-dependent

We selected genes bound by dLsd1 according to our ChIP-Seq dataset and containing a GATA motif in their promoter region to ask whether Serpent or dGATAd binding might influence dLsd1 recruitment. As a first step to assess whether dLsd1 binding to the GATA motif containing targets is dependent on Srp or dGATAd, we depleted either factor using dsRNAs in S2 cells and monitored dLsd1 binding. We chose to start the analysis using S2 cells because *srp* and *dGATAd* depletion was efficient and did not affect *dLsd1* mRNA levels (Supplementary Figure S3A and B). Depletion of *srp* in S2 cell resulted in a reduction in dLsd1 binding in genes containing a GATA motif (*ush*, *fu2*, *SMC1, RpS28b* and *Src64B*) but not in *Pnt*, a dLsd1 target devoid of the GATA motif (Figure 2B). In contrast, dLsd1 binding to the target genes selected was not affected by dGATAd depletion (Supplementary Figure S3C). Taken together, these experiments suggest that Srp facilitates dLsd1 binding to the selected GATA motif containing targets while dGATAd is dispensable.

Consistent with this hypothesis, ChIP experiments using a Srp specific antibody (Supplementary Figure S3D) showed that, in addition to *u-shaped*, a known Srp target (37), Srp also binds a subset of dLsd1 targets containing a GATA motif (Figure 2C). We then asked whether Srp also participates in dLsd1 recruitment *in vivo*. Importantly, the depletion of Srp by RNAi in the ovaries using the *Traffic Jam-Gal4 (TJ-Gal4)* driver (Supplementary Figure S3E) caused a reduction of dLsd1 binding to a subset of GATA-containing targets but not in *Retn*, a dLsd1 target devoid of the GATA motif (Figure 2D). Finally, reciprocal co-immunoprecipitation experiments revealed that GFP tagged dLsd1 can pull down endogenous Srp (Figure 2E) and that GFP tagged Srp can pull down endogenous dLsd1 (Figure 2F). To exclude the possibility that this interaction is indirectly mediated by DNA bridging, benzonase was added to the extract to remove nucleic acids (Supplementary Figure S3F and G). The association of dLsd1 with Srp also occurs in benzonase treated extracts (Figure 2E, F), although benzonase treatment weakens the interaction when the Srp antibody is used to pull down dLsd1 (Figure 2F). Taken together, these data indicate that Srp and dLsd1 can physically interact and can bind a set of common targets.

### Reduced Serpent levels in the ovary result in oogenesis defects

**Figure 3.**
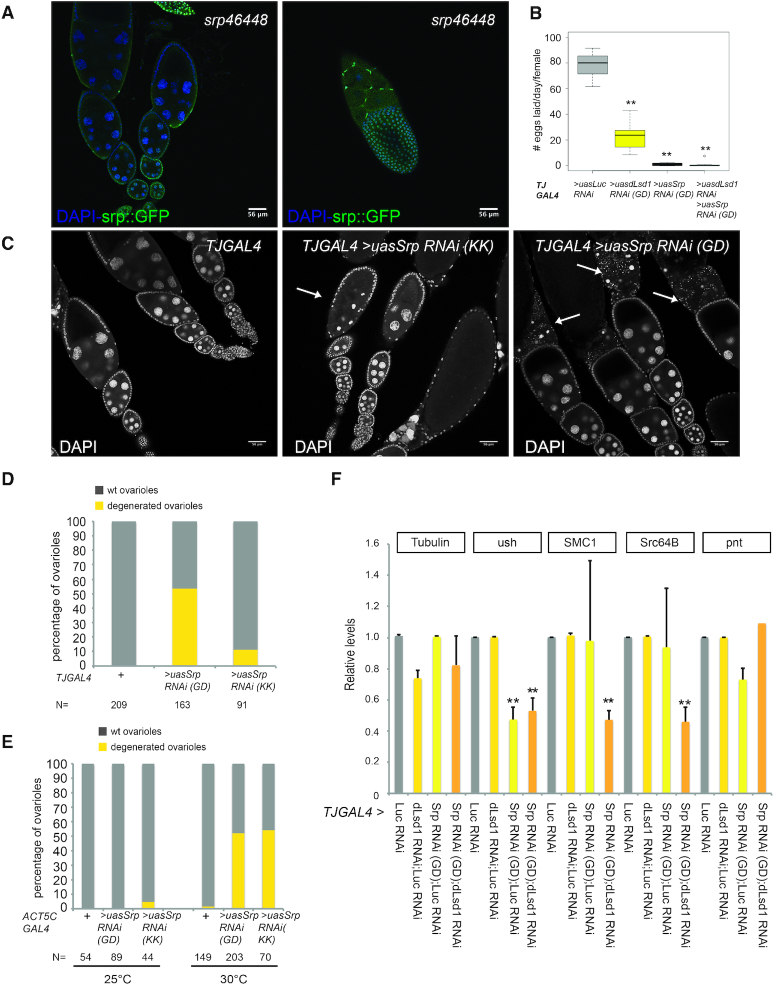
Serpent depletion results in mid-oogenesis defects. (**A**) Serpent::GFP is detected both in follicle and in nurse cells. Ovarioles of *srp46448* flies carrying GFP tagged FlyFOS *srp* were co-stained for GFP (green), and DAPI (blue). (**B**) Flies expressing *srp* RNAi in follicle cells using the *Traffic Jam GAL4* (*TJGAL4*) driver have severe egg laying defects. The numbers of eggs laid per day per female of the indicated genotypes are shown. 10 flies were individually examined for each of the indicated genotypes. A Welch t.test was performed to indicate significance (**P < 0.01). (**C**) Srp depletion by RNAi using the *Traffic-Jam-GAL4* driver results in mid-oogenesis egg chamber degeneration. Ovaries were stained with DAPI. Degenerated egg chambers are indicated by white arrows. (**D, E**) The cumulative percentage of ovariole phenotypes observed in each of the indicated genotypes. N represents the total number of ovarioles scored for each genotype. (**F**) RT-qPCR showing the level of a subset of GATA motif containing target transcripts (ush, SMC1, Src64B) in ovary expressing an RNAi against *srp* and/or *dLsd1* driven by the *TJ-GAL4* driver. The housekeeping gene *Tubulin* and *pnt*, which does not contain the GATA motif are used as controls. A Student's t test was performed to indicate significance (*P < 0.05, **P < 0.01).

Transcriptomics data (Supplementary Table S6) and northern blot analysis (36) show that *srp* is expressed in the ovary. However, little is known about *srp* ovarian expression pattern nor of its function in this tissue. First, we investigated *srp* mRNA distribution in *Drosophila* ovaries by *in situ* hybridization. This analysis documented that *serpent* mRNA is expressed in both germ cells and somatic epithelial cells (Supplementary Figure S4A, B). We also assessed the expression of a GFP tagged Srp transgenic line from the FlyFos library (38). Consistent with our *in situ* hybridization data, this line showed low but detectable Srp expression in the nuclei of both germ cells and somatic epithelial cells, with higher level of expression in follicle cells of late egg chambers stages (Figure 3A, Supplementary Figure S4C, D). These data establish that *srp* is indeed expressed in the ovary.

To investigate the role of Srp in oogenesis, we determined the impact of Srp on egg laying. To do so, we compared the number of eggs laid by females in which Srp was depleted by RNAi using the follicle cell driver *Traffic Jam-GAL4 (TJ- GAL4)* to eggs laid by control flies (*TJ- GAL4 Luc RNAi)* and flies depleted of dLsd1. As expected dLsd1 depleted females laid considerably fewer eggs compared to *TJ- GAL4 Luc RNAi* females, on average 23 per day versus 78 laid by *TJ-GAL4 Luc RNAi* females (Figure 3B). Strikingly, Srp depleted females laid almost no eggs (on average 1 egg per day), revealing a new function for Srp in oogenesis (Figure 3B).

Given the severe egg-laying defect observed in Srp-depleted females, we proceeded to further characterize the effect of Srp depletion in oogenesis with two different RNAi lines targeting *serpent* (from the VDRC GD and KK collections) (Supplementary Figure S3E). Depletion of Srp in follicle cells using the *Traffic Jam-GAL4 (TJ-GAL4)* driver resulted in ovarioles containing degenerate mid-stage egg chambers, as revealed by the pyknotic morphology of the DAPI-stained nuclei (Figure 3C). This phenotype was observed in 53% (*n* = 163) of ovarioles in which Srp was depleted with the GD construct and 11% (*n* = 91) of ovarioles in which Srp was depleted with the KK construct but was never observed in control *TJ-GAL4/+* ovarioles (Figure 3C, D).

To verify if depletion of Srp in germline cells gave similar phenotypes, we used the *Nanos-GAL4* driver. However, *Nanos-GAL4* driven Srp depletion did not result in any egg laying defect (Supplementary Figure S4E) nor in any detectable ovarioles morphology defect using DAPI staining (Supplementary Figure S4F). To confirm the results obtained with the *TJ-GAL4* driver, we deployed the ubiquitous *Act5C-GAL4* driver. However, since the expression of *srp* RNAi using the *Act5C-GAL4* driver resulted in lethality, we temporally restricted RNAi expression by combining *Act5C-GAL4* with a *tub-GAL80^ts^* transgene expressing a temperature sensitive GAL80 inhibitor to block GAL4 activity (39). We kept the flies at a permissive temperature (25°C) until late pupal stages before shifting them to a non-permissive temperature (30°C) for 4 days, then we examined the adult ovaries. We observed the presence of 52% and 54% ovarioles containing degenerate mid-stage egg chambers in flies expressing RNAi for *srp* (*Act5CC-GAL4/+; UAS-srpRNAi (GD)/tub-GAL80^ts^* and *Act5C-GAL4/+; UAS-srpRNAi (KK)/tub-GAL80^ts^*respectively) compared with 1% of ovarioles in control flies (*Act5C-GAL4/+; +/tub-GAL80^ts^)* (Figure 3E). Of note, when the flies were constantly raised at permissive temperature, this phenotype occurred in only 4% of the ovarioles carrying the *UAS-srpRNAi (KK*) transgene and never in control flies or in flies carrying the *UAS-srpRNAi (GD)* transgene (Figure 3E). Since both RNAi lines produced the same phenotype, we consider it unlikely that the observed defect is due to off-target effects. Taken together these results suggest that Srp depletion causes defects in egg chambers culminating in the activation of a mid-stage checkpoint (40) and subsequent degeneration of the affected chambers. The phenotype is not completely penetrant and we do find egg chambers at later stages of development in Srp depleted ovaries (Supplementary Figure S5). However, 28 of the 36 mature eggs observed in Srp depleted ovaries contain shorter dorsal filaments, the respiratory structures of the eggshell (Supplementary Figure S5) versus none of the wild-type counterpart (*N* = 40), indicating that Srp might also control dorsal filaments formation and/or egg maturation.

Having established that Srp depletion causes multiple oogenesis defects, we then tested whether depletion of Srp modifies the phenotypes associated with dLsd1 depletion. *TJ-GAL; uasLsd1 RNAi* ovaries have disorganized ovarioles in which egg chambers feature aberrant numbers of nurse cells and defects in follicle cells patterns (Supplementary Figure S5). Ovaries depleted of both Srp and dLsd1 by RNAi exhibit a stronger phenotype compared with single depletion of Srp or dLsd1, characterized by extremely disorganized ovarioles lacking late stage egg chambers (Supplementary Figure S5). To obtain more quantitative results, we then assessed by RT-qPCR the expression of a subset of genes bound by both Srp and dLsd1 (Figure 2B–D) following Srp and/or dLsd1 depletion in the ovaries. Srp depletion *per se* is sufficient to affect *ush* expression but not sufficient to affect the expression of *SMC1* and *Src64B* (Figure 3F). Interestingly, while dLsd1 RNAi alone does not affect the expression of these targets, we observe a synergistic effect of Srp and dLsd1 on the regulation of *SMC1* and *Src64B*, two common targets (Figure 3F). In summary, we describe a novel and unexpected role for the GATA transcription factor Srp in oogenesis and our results strongly suggest that Srp and dLsd1 cooperate (in the same pathway or in parallel pathways) to regulate key developmental genes implicated in oogenesis.

### Genome-wide expression changes in ovaries depleted of dLsd1

To better define the role of dLsd1 on gene expression in oogenesis, we performed microarray analysis in wild type ovaries and ovaries depleted for dLsd1. To deplete dLsd1, we drove the expression of an RNAi against dLsd1 using the ubiquitous *Act5C-GAL4* driver (Supplementary Figure S6A, B). Comparison of transcripts expression levels between ovaries carrying the *Act5C-GAL4* driver alone and ovaries depleted of dLsd1 revealed 1988 differentially expressed transcripts (log2 fold change > ±0.6, *P* value < 0.05). The transcripts showing significant differential expression levels were about equally divided between those that were up-regulated (1042) or down-regulated (946) (Supplementary Table S7).

**Figure 4.**
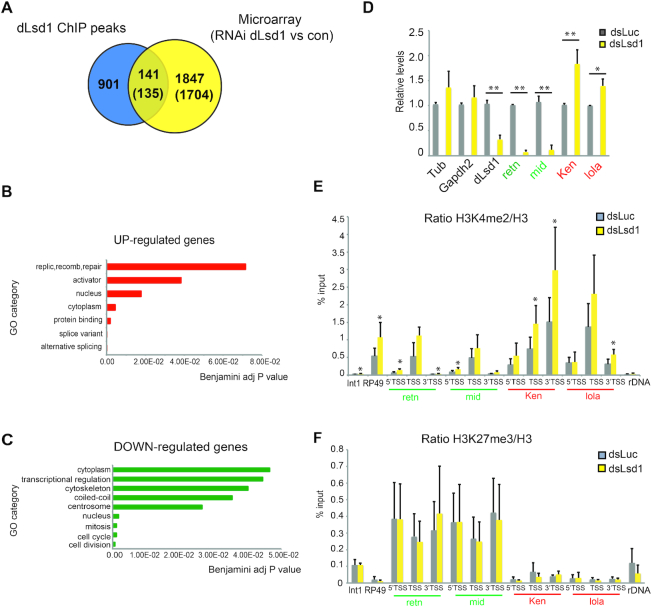
dLsd1 regulates the expression of genes involved in cell cycle and in transcriptional regulation. (**A**) Venn diagram showing the overlap between dLsd1 ChIP-Seq peaks proximal to the TSS and the genes deregulated upon dLsd1 depletion according to the microarray data (log2 fold change >±0.6, *P* value <0.05). The numbers in parenthesis represent the total number of genes containing at least one peak (135), and the total number of genes for which at least one transcript is deregulated (1704). (**B**) Gene Ontology analysis of the genes up-regulated upon dLsd1 depletion and bound by dLsd1. (**C**) Gene Ontology analysis of the genes down-regulated upon dLsd1 depletion and bound by dLsd1. The terms showed in (B) and (C) are the most significantly enriched terms (Benjamini adjusted *P* value <0.1) generated from an analysis with DAVID. (**D**) RT-qPCR analysis of the expression of four dLsd1 targets in S2 cells upon dLsd1 depletion. *Tubulin* and *gapdh2* are two housekeeping genes used as controls. The expression levels are normalized against S2 cells incubated with *dsLuciferase*. Experiments were performed in triplicate and error bars indicate the standard error of the mean. A Wilcoxon test was performed to indicate significance (*P < 0.05, **P < 0.01). (**E, F**) Cross-linked chromatin was isolated from S2 cells incubated with dsRNA against *dLsd1* or *Luciferase* (control), and ChIP analysis was performed using antibodies specific for H3, H3K4me2 and H3K27me3. The ratio between total H3 and each antibody is shown. 5′TSS, TSS and 3′TSS indicate the position of the primers relative to the TSS, with 5′ being upstream and 3′ being downstream of the TSS. Experiments were performed at least in triplicate and error bars indicate standard deviation. A Wilcoxon test was performed to indicate significance (*P < 0.05; **P < 0.01).

To study the most direct effect of dLsd1 occupancy on gene expression, we assessed the relationship between dLsd1 binding and transcriptional changes of the associated targets upon dLsd1 depletion. To do so, we compared our microarrays data to the dLsd1 ChIP-Seq peaks located proximally to promoter regions. We identified 135 transcripts that showed differential expression and that had at least one dLsd1 peak located in their promoter and/or gene body (some targets had multiple dLsd1 peaks, as we identified a total of 141 dLsd1 peaks) (Figure 4A) (Supplementary Table S8). We found a positive correlation between dLsd1 binding and expression changes (*P* value = 0.012, Pearson's Chi-squared test). Of these 135 target transcripts, 53 were up-regulated and 82 were down-regulated upon dLsd1 depletion. Gene ontology analysis of the up-regulated genes included the term replication, recombination and repair (Figure 4B). Two of the most enriched gene ontology categories of the down-regulated genes were cell division and cell cycle, suggesting that dLsd1 contributes to cell proliferation like its mammalian counterpart (Figure 4C). The peak location may not be a discriminating feature distinguishing up-regulated and down-regulated genes, as more than 70% of peaks are located in the promoter of the de-regulated genes, regardless of the fact that the gene is up-regulated or down-regulated (Supplementary Table S8). Given the interplay between dLsd1 and Srp, we looked for the presence of the CGATA motif at dLsd1 direct targets. We found the exact CGATA motif in the majority of them (64%), of these 68% are down-regulated upon dLsd1 depletion (Supplementary Table S8), indicating that dLsd1 in most cases positively regulates their expression (*P* value = 0.002, Pearson's Chi-squared test), possibly in conjunction with Srp. In agreement with the microarray results, we confirmed by RT-qPCR that dLsd1 depletion resulted in a significant decrease in expression of *retn*, *mid* and *Src64B* and in a significant increase in expression of *Ken* and *CHES-1 like*, without affecting the expression of the housekeeping genes *α-tubulin* and *gapdh2* (Supplementary Figure S6B).

To examine the relationship between presence of dLsd1, histone marks and gene transcription, we selected two dLsd1 bound genes (Supplementary Figure S1) that are up-regulated (*Ken* and *lola*) and two that are down-regulated (*retn*, and *mid*) in response to dLsd1 depletion according to our microarray analysis. We then performed ChIP against activating and repressive histone marks upon dLsd1 depletion. We performed these experiments in S2 treated with a dsRNA targeting either *Luciferase* or *dLsd1* to have a more homogeneous cell population as well as a reproducible and drastic reduction of dLsd1 levels (Supplementary Figure S6C). As expected, dLsd1 reduction was accompanied by a global increase in H3K4me2 and H3K4me1 levels, while the levels of H3K4me3 were unaffected (Supplementary Figure S6C). In agreement with the microarray results, we found by RT-qPCR experiments that dLsd1 depletion resulted in a significant decrease in the expression of *retn* and *mid* and in a significant increase in expression of *Ken* and *lola* (Figure 4D). We then performed ChIP for H3K4me2, me3, H3K9me2 and H3K27me3 in S2 cells treated with dsRNA against *Luciferase* or *dLsd1*. The ChIP results show that depletion of dLsd1 resulted in an increase of the levels of H3K4me2 at the target promoters regardless of the transcriptional output (Figure 4E) whereas H3K4me3, H3K9me2 and H3K27me3 levels were not affected by dLsd1 depletion (Figure 4F and Supplementary Figure S6D, E).

Taken together, these results show that dLsd1 regulates directly and indirectly hundreds of genes in the ovary.

### dLsd1 depletion results in up-regulation of transposons

**Figure 5.**
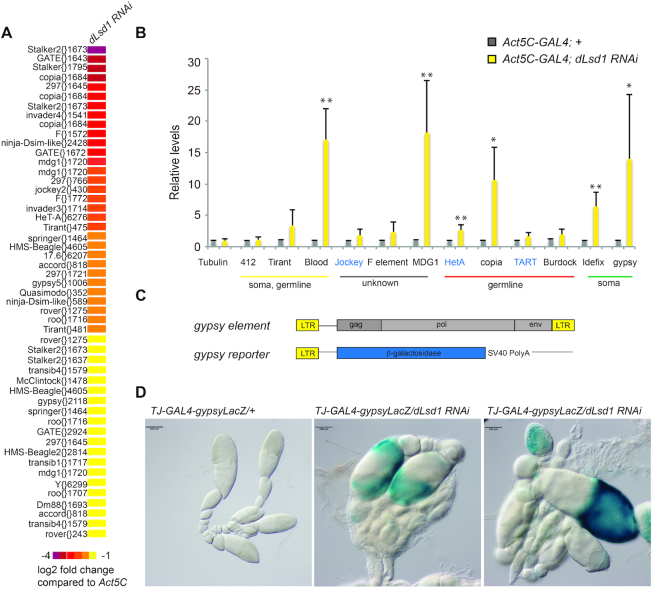
dLsd1 depletion in the ovary results in de-repression of transposable elements. (**A**) Heatmap summarizing the transposon de-repression observed by microarray analysis upon dLsd1 RNAi in the ovary. *Act5C-GAL4* was used to drive *dLsd1* RNAi. The log_2_ fold change is calculated as follow: intensity in log_2_ of control condition—intensity in log_2_ of *dLsd1 RNAi* condition. (**B**) RT-qPCR analysis of the levels of expression of a subset of transposons in the ovary upon ubiquitous dLsd1 depletion. A Welch t-test was performed to indicate significance (*P < 0.05; **P < 0.01). (**C**) Schematic representation of the gypsy element and of the gypsy reporter. (**D**) β-gal staining of ovarioles carrying a *gypsy-lacZ* reporter and a *Traffic-Jam-GAL4* driver in the presence or absence of *dLsd1*-RNAi.

Given the previously discovered dLsd1 function in regulating heterochromatin homeostasis (13,41,42), we next asked whether its depletion may alter the transcription of transposable elements, which mainly reside in heterochromatin domains (43). The genome of *Drosophila melanogaster* harbors diverse families of transposable elements (TE) (44); we aligned the sequences of the microarray probes that did not align to gene transcripts to the Dm6 Transposable elements sequences and found up-regulation of transcripts from 22 families of long terminal repeats (LTR) containing TEs, 3 families of LINE-type TEs, and two families of DNA-type TEs (Figure 5A). To confirm the de-repression of transposons in ovaries depleted by dLsd1, we performed quantitative real time PCR (qPCR) on a subset of TEs (*Tirant, F-element, MDG1, HetA, copia and gypsy*) that were found to be up-regulated in the microarray (log_2_FC > 0.6, *P* value < 0.05) and on some other well-characterized transposon families. These transposons can be grouped in two cohorts, depending on whether piRNA targeting them are primarily found in germline cells or somatic cells, plus an additional cohort grouping TEs for which this information is missing (unknown) (Figure 5B). Transcripts from *MDG1*, *HetA*, *copia*, *Blood* and *Idefix* and *gypsy* elements were significantly elevated in dLsd1-depleted ovaries compared to wild-type ovaries (Figure 5B). At least one element in each cohort was de-silenced by depletion of dLsd1, indicating that dLsd1 globally controls TE silencing.

To further confirm the de-repression of TEs observed upon dLsd1 depletion, we used a transgenic line carrying a *gyspy-LacZ* reporter construct (Figure 5C and (45)). This construct expresses β-galactosidase under the control of gypsy LTR. In agreement with the microarray and RT-qPCR results, dLsd1 depletion by RNAi in follicle cells using the *TrafficJam-GAL4* driver resulted in de-silencing of the reporter (Figure 5D). Taken together, these data show that dLsd1 depletion globally affects TEs silencing.

**Figure 6.**
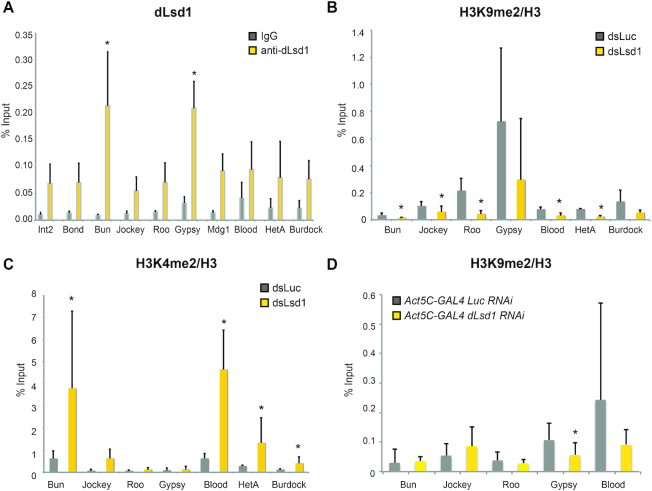
Reducing dLsd1 levels results in an increase in H3K4me2 and a decrease of H3K9me2 at TEs. (**A**) ChIP-qPCR analysis of dLsd1 binding at promoters or 5′UTR of a set of transposons in the ovary. (**B, C**) ChIP-qPCR analysis showing the H3K9me2/H3 ratio (B) and H3K4me2/H3 ratio (C) at promoters or 5′UTR of a set of transposons upon dLsd1 depletion in S2 cells. All experiments were performed at least in triplicate except for the measure of *Gypsy* H3K4me2 levels, which was done in duplicate. (**D**) ChIP-qPCR analysis of H3K9me2/H3 ratio at promoters or 5′UTR of a set of transposons in ovaries expressing RNAi against *Luciferase (Luc)* compared to ovaries expressing *dLsd1* RNAi. Error bars indicate standard deviation of the means. A Wilcoxon test was performed to indicate significance (*P < 0.05).

Visual analysis of dLsd1 ChIP-Seq tracks suggested that dLsd1 could directly bind TEs (Supplementary Figure S7A). While repetitive regions can be covered in high and medium coverage ChIP-seq experiments (46) some repetitive regions might be underrepresented in our analysis as multi-mapping repetitive sequences were retained only once. Therefore, we decided to more extensively analyze the signal at these regions by mapping dLsd1 ChIP-Seq, pre-immune and input control reads to the annotated repeats elements of *Drosophila melanogaster*. This analysis revealed a small enrichment of the dLsd1 signal at a subset of simple repeats (FDR<0.05) (Supplementary Figure S7B). In parallel, we performed ChIP-qPCR assays in the ovary, and found that the TSS of Gypsy was significantly bound by dLsd1, while we did not detect a significant enrichment at the other TEs tested (Figure 6A). We then verified whether dLsd1 was directly regulating the main known regulator of transposons silencing (47). We found dLsd1 peaks only in five of the 41 annotated regulators of transposons silencing (*minotaur*, *asterix*, *tud*, *CG13741* and *tej)*. However, the expression of these genes was not altered upon dLsd1 depletion in the ovary, according to the microarray dataset and to RT-qPCR (Supplementary Figure S7C), suggesting that dLsd1 does not affect transposon expression indirectly, by regulating the expression of components of the piRNA pathways.

The Drosophila genome harbors more than 130 different family of transposons and the majority of them are located in pericentric or telomeric heterochromatin (48). These TEs are silenced and are enriched in H3K9me2 marks. Importantly, dLsd1 has been shown to affect heterochromatin homeostasis (13,41,42). To determine whether dLsd1 depletion affects histone methylation at transposons, we performed ChIP experiments in S2 cells depleted of dLsd1. Interestingly, we observe a marked decrease of the heterochromatic H3K9me2 marks and an increase of H3K4me2 at a subset of TEs when we deplete dLsd1 (Figure 6B, C). Importantly, we confirmed this tendency toward a decrease in K9me2 marks at TE loci in ovary samples depleted of dLsd1 by RNAi (Figure 6D). These results correlate with the observed de-silencing of TEs upon dLsd1 depletion and suggest that dLsd1 could be an important player of the chromatin-based suppression mechanism of TEs.

In summary, our analysis shows that dLsd1 plays a global role in TE silencing by modulating the levels of H3K4me2 and H3K9me3 at transposons loci.

### dLsd1 and Piwi genetically interact

TEs are normally kept silenced in the Drosophila gonads by the piRNA pathway and depletion of piRNA pathway components result in oogenesis defects and sterility (49).

**Figure 7.**
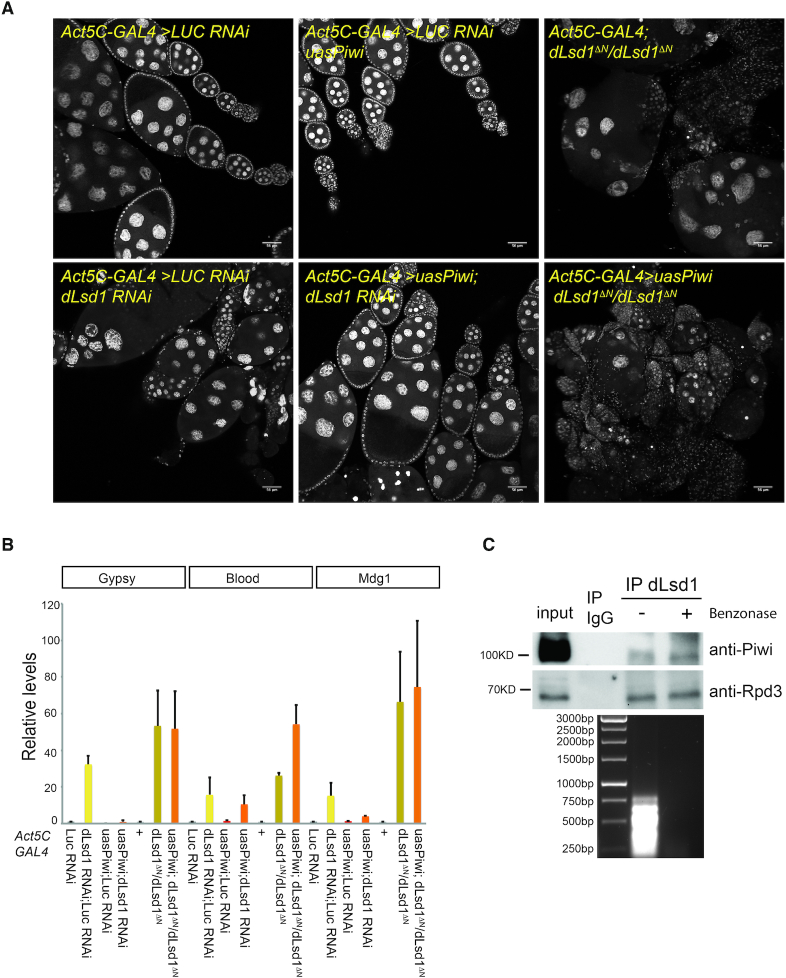
Piwi requires dLsd1 to repress TEs. (**A**) *dLsd1* genetically interacts with *Piwi*. Images of DAPI stained ovarioles from the indicated genotypes. (**B**) RT-qPCR analysis of the levels of expression of a subset of transposons in ovaries of the indicated genotypes. RT-qPCR was performed in biological duplicates. Error bars indicate standard deviation of the means. (**C**) dLsd1 physically interacts with Piwi. Immunoblot showing co-immunoprecipitation of endogenous Piwi and dLsd1 in ovaries dissected from wild type (*w^1118^*) flies. Rpd3, a core component of the dLsd1 complex, was used as a positive control. Benzonase was added where indicated. DNA digestion upon Benzonase treatment was monitored by agarose gel electrophoresis.

Therefore, we reasoned that de-repression of transposons might at least partly explain the oogenesis defects observed upon dLsd1 depletion (13). Hence, we tested whether ectopic expression of one of the key component of the TE silencing machinery, Piwi (P-element induced wimpy testis) can rescue the phenotypes associated with dLsd1 loss of function. To do so, we ectopically expressed Piwi in a dLsd1-depleted background. Partial dLsd1 depletion by RNAi using the ubiquitous *Act5C-GAL4* driver results in disorganized ovarioles structures (Figure 7A). As previously shown, *dLsd1* null ovaries (*dLsd1ΔN/dLsd1ΔN*) (13) fail to develop past the initial stages of oogenesis (Figure 7A). Ectopic expression of Piwi can almost completely rescue the defects due to partial depletion of dLsd1 but fails to rescue the defects associated to *dLsd1* null mutation (Figure 7A, Supplementary Figure S8A). These results suggest that Piwi requires dLsd1 to repress TEs in the ovary.

We then examined the level of a subset of TEs by RT-qPCR. Null mutation of *dLsd1* results in a robust de-repression of TEs, confirming the results obtained by dLsd1 RNAi (Figures 5B and 7B), while ectopic expression of Piwi results in silencing of a subset of TEs (Supplementary Figure S8B). In addition, we observe that the dLsd1 dependent de-repression of Gypsy, Blood and Mdg1 is rescued by ectopic expression of Piwi when some residual dLsd1 is present, but not in a complete loss of function background (Figure 7B, Supplementary Figure S8C).

Having established that dLsd1 and Piwi genetically interact, we asked whether they also physically associate. To do so, we performed reciprocal co-immunoprecipitation experiments and found that endogenous dLsd1 can pull down Piwi in ovaries and vice versa, indicating that Piwi and dLsd1 can physically interact (Figure 7C, Supplementary Figure S8D). Although this interaction is weak, treatment with Benzonase did not prevent it, indicating that the association between dLsd1 and Piwi is not solely dependent on nucleic acids (Figure 7C, Supplementary Figure S8D).

## DISCUSSION

Previous studies have established that *dLsd1* mutation causes severe oogenesis defects in Drosophila. Here, we provide a comprehensive map of endogenous dLsd1 binding sites in the genome of ovarian cells. We discover an unexpected interplay between dLsd1 and the GATA transcription factors Serpent and through transcriptomics analysis, we provide evidence that dLsd1 plays a dual function in the ovary: regulating specific genes and silencing transposable elements.

Specifically, we report that dLsd1 localizes at the transcriptional start sites of developmental genes. Interestingly, many dLsd1 targets are transcribed and marked by H3K4me2 and Pol2-Ser5. Our analysis of the ChIP-Seq data also shows that dLsd1 peaks are discrete, supporting a model in which dLsd1 is recruited locally by transcription factors. Motif analysis of these peaks unveiled a list of transcription factors that could guide dLsd1 to specific set of targets. Therefore, this list can be used to explore new mechanisms of dLsd1 recruitment. In particular, more than one third of dLsd1 peaks carry a motif recognized by GATA transcription factors. We show that dLsd1 and the GATA transcription factor Serpent co-occupy a subset of GATA motif containing promoters and that dLsd1 and Serpent can physically interact, it remains to be determined whether this interaction is direct or indirect. Importantly, we provide evidence that Serpent plays a role in recruiting dLsd1 to GATA motif containing promoters. Previous studies have shown that Serpent plays a role in organogenesis (50), however a role for this transcription factors in oogenesis had not been described. Here, we show that Serpent depletion results in multiple oogenesis defects, opening the road to a more detailed analysis of its function in this developmental context.

Comparing the transcriptional profile dataset of dLsd1 depleted ovaries to the ChIP-Seq dataset revealed direct dLsd1 targets. These targets include genes involved in cell cycle, transcription, recombination and repair. Some of these genes have already been implicated in oogenesis, including the RNA binding protein Sex-lethal (Sxl), which has been implicated in cystoblast differentiation (51), or the transcription factor midline (mid), which together with longitudinal lacking (lola), has been shown to play a role in gonad formation (52,53). Also *src64*, which encodes for a tyrosin kinase implicated in actin dynamics in oogenesis (54), is bound by dLsd1 and down-regulated upon dLsd1 depletion. Their de-regulation could partially explain some of the phenotypes observed in *dLsd1* mutant ovaries. Others targets have not been previously identified as being important for oogenesis and could thus be tested for their biological role in this tissue. These datasets therefore provide an important resource for elucidating novel oogenesis regulatory mechanisms.

As reported previously in mammalian cells, dLsd1 depletion in the ovary results both in down-regulation and up-regulation of target gene expression, indicating the dLsd1 acts both as a co-repressor and as a co-activator. Aside from direct targets, we found that 1847 mis-regulated transcripts were not bound by dLsd1 according to our ChIP-Seq dataset. This could be due to the fact that the transcriptional changes observed upon dLsd1 depletion in the ovary are associated with an aberrant developmental program or that dLsd1 control the expression of these targets from distal enhancers. Conversely, 902 peaks are located in genes that are bound by dLsd1 but whose transcription is not affected by its depletion. There are several potential explanation for this lack of effect, the most obvious being the presence of compensatory mechanisms or the fact that the partial depletion observed in the RNAi line is not sufficient to completely titrate dLsd1 out of the high affinity targets. Alternatively, dLsd1 depletion has no effect because the transcription factors required for the activation of a subset of targets are not expressed in the ovary. Similarly, dLsd1 might be bound to the gene but be kept in an inactive state by the presence of an inhibiting factor or histone mark and therefore its absence would not cause any effect. This has been postulated to be the case in embryonic stem cells (ESC) where high levels of acetylated histone block LSD1 demethylase activity and only when histone acetyl tranferases levels decrease during cell differentiation, LSD1 becomes active and can repress transcription (4). Finally, we cannot exclude that dLsd1 does not play a role in the transcription of these genes but is implicated in other functions (e.g.: replication, chromatin conformation, repair) or that we missed the effect on expression because they occur in a small cell population.

Consistent with previously published data, we detect a reproducible increase in H3K4me2 levels at the regulatory regions of dLsd1 targets activated upon dLsd1 depletion. Intriguingly, we also observe a small but significant increase in H3K4me2 levels at genes that are repressed by dLsd1 depletion. Whether K4 methylation and/or dLsd1 catalytic activity are required for its function at targets are open questions. A detailed analysis of dLsd1 catalytic mutants as well as the identification of dLsd1 non-histone substrates will be necessary to thoroughly address them. Regardless, our analysis of the relationship between chromatin marks, dLsd1 occupancy and transcription suggests that the pre-existing chromatin environment is likely to make an important contribution to the transcriptional response of a gene to dLsd1 depletion. We find that two genes that are lowly transcribed and feature low levels of H3K4me2/me3 and high levels of the H3K27 mark (*retn* and *mid*) are down-regulated upon dLsd1 depletion. While *Ken* and *lola*, which are expressed in the ovary and feature high levels of H3K4me2/me3 and low levels of the H3K27 mark are up-regulated upon dLsd1 depletion. It is therefore tempting to speculate that dLsd1 acts as a modulator of transcription dampening the level of expression of a gene in an activating chromatin context, while in a repressive chromatin context, it poises the genes for activation. Interestingly, the hypothesis that dLsd1 fine-tunes transcription rather than acting as an off switch is consistent with our analysis of the ChIP-Seq results showing that the majority of dLsd1 peaks are located at transcribed genes and with the growing evidence that fine-tuning of gene expression is a common mechanism by which many co-repressors function (55). Single-cell RNA-Seq analysis in dLsd1-depleted cells compared to wild-type will be needed to further elucidate the precise contribution of dLsd1 in the transcription of its targets.

In addition to its function at protein coding genes, we found that dLsd1 strongly silence transposable elements transcription. Genome-wide screen aimed at finding novel regulators of transposon silencing have been performed in the past and resulted in the identification of many factors involved in the transcriptional silencing of transposons. Mining these datasets, we found dLsd1 among the factors identified by Czeck et al (56), while dLsd1 depletion did not affect transposon silencing in the screen published by Handler et al (57). These results are contradictory and no detailed analysis of dLsd1 role in TE silencing has previously been reported. In a recent study, the Hannon team identified Panoramix as a critical regulator of transposons and showed that dLsd1 depletion impaired Panoramix-dependent repression of a reporter line (58), hinting at a role for dLsd1 in the suppression of transposons. This hypothesis was very recently confirmed by Yang et al, who reports de-repression of a subset of TEs upon dLsd1 germline-specific knockdown (59). Our data support and reinforce the evidence of an important role for dLsd1 in TE silencing not only in the germline, but also in somatic cells. Here we show through multiple assays that silencing of several transposable elements (TE) is impaired by dLsd1 depletion. Furthermore, we show that the increased TE expression observed upon dLsd1 depletion is paralleled by a decrease in the repressive H3K9 methylation mark and an increase in the activating H3K4 methylation mark. Our observations suggest the existence of a dLsd1 dependent chromatin-based transposon silencing mechanism. This mechanism is most likely crucial for TE repression, as our results show that ectopic Piwi expression cannot rescue the TE de-silencing and ovaries defects associated to a null *dLsd1* background, suggesting the dLsd1 is required for Piwi dependent TE silencing. Consistently, we report a weak but reproducible interaction between dLsd1 and Piwi. It would be important to determine whether this interaction is direct or if it is mediated by additional chromatin factors. Together these results suggest the existence of a chromatin-based transposon silencing mechanism dependent on the interaction between dLsd1 and Piwi and open the road to the study of the interplay between LSD1 and Piwi in vertebrates.

The majority of transposons are located in pericentric or telomeric heterochromatin (48), and it had been previously shown that dLsd1 is a strong suppressor of variegation and promotes heterochromatin formation at the boundary between heterochromatin and euchromatin (13,41,42). It is possible that the effect of dLsd1 on the expression of the transposons located in heterochromatin might be indirectly due to dLsd1 role in the control of heterochromatin structure. This hypothesis could explain the lack of enriched dLsd1 peaks at TEs in our dataset. In alternative, dLsd1 might bind TEs at low levels or transiently. Furthermore, we cannot exclude the possibility that the lack of enrichment is due to the inherent difficulty in shearing heterochromatin and in sequencing or mapping repetitive sequences (60,61). The recently developed Gradient-Seq technique, which allows enriching for sonication-resistant heterochromatin (62) coupled with RNA-Seq for small RNAs could be used to test if dLsd1 regulates TEs directly by binding to their regulatory elements or indirectly through deregulation of piRNAs.

Regulation of TEs by LSD1 seems to be a conserved mechanism, since LSD1 was found to regulate transposable elements in mice (63,64) and very recently also in humans (65). Importantly in human cells, reactivation of TE elements upon dLsd1 inhibition triggers an immune response that renders cancer cells more susceptible to immunotherapy (65). In mice LSD1 null oocytes give rise to zygotes that arrest by the two cells stage and this arrest is accompanied by perturbation in the expression of genes and retrotransposons (64). This striking similarity to our results is suggestive of a highly important and conserved function of LSD1, which could be investigated in more details by further exploring the interplay with Piwi that we unveiled. Interestingly, a large number of cellular genes have promoters or enhancers derived from ancient retroviral insertions and Macfarlan and colleagues suggests that LSD1 could use ancient retroviral insertion to suppress gene expression (63). It would be interesting to test whether in Drosophila dLsd1 has been co-opted to regulate cellular genes with promoters derived from retroviral elements.

In summary, we propose that LSD1 has an evolutionary conserved role in the protection from excessive endogenous retrotransposons activity and in the regulation of key developmental genes. Importantly, the identification of Serpent at a subset of dLsd1 target genes opens the road to a more detailed study of the interplay between dLsd1 and Serpent in oogenesis. The identification of other dLsd1 partners implicated into its targeting to specific genomic regions will also help elucidate the role of demethylating complexes in oogenesis. Given the high level of conservation of LSD1 from Drosophila to mammals, equivalent links between dLsd1 and Srp and between dLsd1 and Piwi are likely to exist in mammals and could be studied to determine their contribution to the many diseases associated to aberrant LSD1 activity.

## DATA AVAILABILITY

ChIP-Seq and microarray data performed in this study are publicly available at NCBI (GEO accession numbers: GSE110331, GSE110751). ChIP-Seq tracks uploaded to the UCSC genome browser can be viewed using the following link: http://genome.ucsc.edu/s/ldistefano/dm6_LDiStefanoData.

## Supplementary Material

gkz1142_Supplemental_Files
