# Supplementary material for: A dual role of dLsd1 in oogenesis: regulating developmental genes and repressing transposons
Source: Nucleic Acids Res. 2019 Dec 4;48(3):1206–24. doi: 10.1093/nar/gkz1142 (PMC7026653; doi:10.1093/nar/gkz1142)
Supplement: gkz1142_Supplemental_Files [file gkz1142_supplemental_files.zip › Supplemental Files Nov 2019.pdf]

SUPPLEMENTARY DATA

SUPPLEMENTARY FIGURE LEGENDS

Supplementary Figure S1. Screenshots of dLsd1 peaks. A) Integrated Genome Browser (IGB) screenshots of dLsd1 enriched sites. Anti-dLsd1, pre-immune serum IP and input are shown.

Supplementary Figure S2. dLsd1 is enriched at gene networks important for development. A) dLsd1 peaks size distribution. Histogram showing the width frequency of dLsd1 peaks in ovaries. The width frequency of dLsd1 peaks defined using pre-immune serum IP as control (pink) is shown. Similar results were obtained using input as control (blue) or taking the genes in common between the two normalizations (union in yellow). **B)** dLsd1 peaks are located at or in close proximity of the TSS. dLsd1 binding around transcriptional start sites is shown. **C)** GO analysis of the genes associated to dLsd1 peaks. The terms showed are the most significantly enriched terms (Bonferroni adjusted p value <0.01) generated from an analysis with DAVID.

Supplementary Figure S3. dGATAd depletion does not affect dLsd1 binding. A) RT-qPCR showing the relative level of expression of *serpent* and *dLsd1* upon depletion of *serpent* in S2 cells. *Tub* and *GAPDH2* are two housekeeping genes. A Student's t test was performed to indicate significance (**, P < 0.01). **B)** RT-qPCR showing the relative level of expression of *dGATAd* and *dLsd1* upon depletion of *dGATAd* in S2 cells. A Student's t test was performed to indicate significance (**, P < 0.01). **C)** ChIP-qPCR analysis of dLsd1 binding at a subset of dLsd1 targets carrying a GATA motif in S2 cells treated with either dsRNA for Luciferase (control) or dsRNA for dGATAd. A Wilcoxon test was performed to indicate significance (*, P < 0.05, **, P < 0.01, n.s, non significant). **D)** Immunoblots of Kc167 cell lysates incubated with dsRNA against *srp* or *GFP* were probed with antibodies against Srp and Tubulin. **E)** RT-qPCR showing the relative level of expression of *serpent* and *dLsd1* in ovaries expressing RNAi targeting *srp* under the control of the Traffic Jam-GAL4 driver. A Student's t test was performed to indicate significance (*, P< 0.05; **, P < 0.01). **F, G)** Agarose gels stained with Ethidium bromide to evaluate DNA digestion in S2 extracts treated with Benzonase (Ben) compared to untreated samples. M stands for molecular weight marker.

Supplementary Figure S4. Srp expression in the ovary. A, A') Ovarioles hybridized *in situ* with either an antisense probe for *lacZ* mRNA (A) or *srp* mRNA (A'). DAPI staining shows DNA. **B, B')** An ovariole with later stage egg chambers hybridized *in situ* with an antisense probe for *srp* mRNA. **C, D)** Ovarioles

of *srp46448* flies carrying GFP-tagged FlyFOS *srp* were co-stained for GFP (green), phalloidin (red) and DAPI (blue). Panels **C, C'** show Srp expression pattern in a later stage egg chamber. Panels **D, D'** show Srp expression pattern in the germarium and in the early stage egg chambers. **E)** Box plot representing the median number of eggs laid per female per day by females of the indicated genotypes. N indicates the number of females used for each genotype. **F, F', F'')** DAPI staining of ovarioles expressing RNAi against *Luciferase* (F) or *srp* (F',F'') under the control of the Nanos GAL4 driver (*NOS-GAL4*).

Supplementary Figure S5. Double depletion of Srp and dLsd1 results in a stronger phenotype compared to single depletion of Srp or dLsd1. DAPI staining of ovaries of the indicated genotypes. Arrows indicate late stage egg chambers with normal (*TJ-GAL4>Luc RNAi*) or shorter dorsal filaments (*TJ-GAL4> Srp RNAi)*.

Supplementary Figure S6. Effects of dLsd1 depletion on targets transcription and chromatin marks. A) Western blot showing dLsd1 protein levels in ovary expressing an RNAi against *dLsd1* driven by the *Act5C-GAL4* driver. Tubulin was used as loading control. **B)** RT-qPCR showing *dLsd1* mRNA levels and the level of a subset of target transcripts in ovary expressing an RNAi against *dLsd1* driven by the *Act5C-GAL4* driver. *GAPDH2* and *Tubulin* are two housekeeping genes used as controls. A Student's t test was performed to indicate significance (*, $P < 0.05$, **, $P < 0.01$). **C)** Immunoblots of S2 cell lysates incubated with dsRNA for *dLsd1* or with dsRNA for *Luciferase* were probed with antibodies against dLsd1, H3K4me1, H3K4me2, H3K4me3. Tubulin was used as a loading control. **D), E)** Cross-linked chromatin was isolated from S2 cells incubated with dsRNA against *dLsd1* or *Luciferase* (control), and ChIP analysis was performed using antibodies specific for H3, H3K4me3, and H3K9me2. The ratio between each antibody and total H3 is shown. ChIP data are the result of three independent immuno-precipitations. Error bars indicate standard deviation. A Wilcoxon test was performed to indicate significance (*, $P < 0.05$; **, $P < 0.01$).

Supplementary Figure S7. Association of dLsd1 with repeat classes and effect of dLsd1 depletion on the expression of components of the piRNA pathways. A) Screeshot of University of California Santa Cruz (UCSC) Genome Browser showing dLsd1 ChIP-Seq peaks located proximally to a TE of the Gypsy family (TIRANT). **B)** Box plots showing the distribution of fold changes (in log scale) of read counts at simple repeats. The median between experimental replicates is shown. When filtering by FDR <= 0.05, we obtained a significant dLsd1 enrichment at 46 repeats (dLsd1 vs input) and 64 repeats (dLsd1 vs pre-immune) belonging to the 'Simple repeats' class. **C)** RT-qPCR showing the relative level of expression of

the indicated genes upon depletion of *dLsd1* by RNAi in the ovary using the *Act5C-GAL4* driver. *Tub* is an housekeeping gene used as control while *Tejas*, *CG13741*, *Minotaur*, *Asterix* and *Tudor* are known components of the piRNA pathway. Experiments were performed in triplicates and standard deviation is shown. A Student's t test was performed to indicate significance (*, P < 0.05; **, P < 0.01).

Supplementary Figure S8. Ectopic Piwi expression results in repression of a subset of TEs. A) Images of the same ovaries as in Figure 7A. Tomato is used as a read-out of the *Act5C-GAL4* driven expression of the uas-Tomato-Piwi transgene. **B)** RT-qPCR showing the relative level of expression of the indicated TEs upon ectopic expression of Piwi in the ovary using the *Act5C-GAL4* driver. Experiments were performed in triplicates and standard deviation is shown. A Student's t test was performed to indicate significance (*, P< 0.05; **, P < 0.01). **C)** RT-qPCR analysis of the levels of expression of *Tubulin*, *dLsd1*, *piwi* in ovaries of the indicated genotypes. RT-qPCR was performed in biological duplicates. Error bars indicate standard deviation of the means. **D)** Immunoblot showing co-immunoprecipitation of endogenous dLsd1 with a Piwi antibody in OSS cells. Tubulin was used as negative control and the immunoprecipitated Piwi is shown. Benzonase (Ben) was added where indicated and an agarose gel showing DNA digestion in the presence of benzonase is shown.

SUPPLEMENTARY TABLES

Supplementary Table S1. Genomic coordinates of dLsd1 peaks normalized against the pre-immune IP (xlsx)

Supplementary Table S2. List of primer set sequences used for ChIP

Target	Primer name	Sequence 5'-3'	Reference
Int	IntF	TCTATCCGCTGCGGGAAATC	(1)
	IntR	TTTCGGAGTTGAGTCGACCG	(1)
	lolaUpTSSF	TCCCGACTGGGGAAGTAGA	this study
	lolaUpTSSR	ATTGCTAATGCAAGGCGGTG	this study
lola	lolaTSS	GGTGTTTTTCCAATCGGTGCT	this study
	lolaTSSR	TCTCGTTGCTTTTCCACCCA	this study
	lolaDownTSSF	ATCTCTCGCTAACAGCACGG	this study
	lolaDownTSSR	CCCTCTCACTCGCTCCTTTT	this study
	KenUpTSSF	CCTCCGCGCAGAGACAGTTAC	this study
	KenUpTSSR	GTGTCCTGGTCCTGGTTCTG	this study
Ken	KenTSSF	TCGATACATCGACCGAGCAG	this study
	KenTSSR	TGCTTTTGCCTTGCTTTGCT	this study
	KenDownTSSF	TGCCATTTTCCCTCGCTTCT	this study

	KenDownTSSR	CGTTTTCCTGCCCTCTCTGT	this study
mid	midUpTSSF	ACATGCCCACTTTGTCGCTA	this study
	midUpTSSR	CGGAAATCCTGCGATCCTGA	this study
	midTSSF	ATAGAGAGGTGCGACCCACAA	this study
	midTSSR	TGAACGTGCGACAGAGAGAAA	this study
	midDownTSSF	ACTTGCAGCGCTCTTTTGTG	this study
	midDownTSSR	CGGCCGTCGCTAATTTTCTG	this study
retn	retnUpTSSF	GGCGGCGAAAAATGGAAAGT	this study
	retnUpTSSR	AATTCGTGGGGCAAAATGGC	this study
	retnTSSF	CGTATTGCGCACACTCTCAC	this study
	retnTSSR	TCGAACGAATGGAATTGCACG	this study
	retnDownTSSF	TCTGTACAGCCAACGCCATT	this study
	retnDownTSSR	AACGCGATTTCATTCGCTCG	this study
Rp49	RP49F	CCAGGTTGGGCTAGCTTCTTG	(1)
	RP49R	CCTGTGAGAGTTCGCCAAATG	(1)
CHES-1-like	CHES-1P1F	CGCGCCAATTAAGTGCTACC	this study
	CHES-1P1R	ACAACAAATTAGCGTCTCGGT	this study
Bun	BunF	CGATGGCAAGCCAACAACAA	(1)
	BunR	TGCAATTTTCTCAACGCGCA	(1)
Ftz-f1	Ftz P1F	CGAACGGGAACACAACACAC	this study
	FtzP1R	ATGCTGCCTCTCTGTCCTTG	this study
Sxl	SxlF	TCTGCGTGTTTTCCACTATCG	this study
	SxlR	AACAATACACACACAGACACTGC	this study
RpS28b	RpS28bF	AGGCATACGGAGGGTAAAAACT	this study
	RpS28bR	TTGACAGCCGCAAGCAAAAA	this study
RpL3	RpL3F	AAGAGAACAGACATGGTGAGCA	this study
	RpL3R	AACACGTGGATGTTGCAAGT	this study
Mnt	MntF	GAGAGAGAGCGACATCTGCAT	this study
	MntR	CAGCTTCGCCTAAATCACGC	this study
rdx	rdxp2F	GCGAGTTTCACCGAAGCAAT	this study
	rdxp2R	CATAAAGTCGCGTTCCGCTG	this study
Src64B	Src64BF	ATAAGAAGCCGCCGAAAAACG	this study
	Src64BR	GGTGCATTCCCTCTCTCTCTT	this study
fu2	fu2F	CAGCGGCTTATCGATTTCCTC	this study
	fu2r	AGCCACACCGGTAGCAAATAA	this study
pnt	PNTF	AACAAGATCCACTCGTCGCT	this study
	PNTR	TTCATTCATACTCGCCAGGCA	this study
Imp	Impf	CGCAATCGATCTCGGGACA	this study
	ImpR	AAACTCGCTAAACGCAACAGT	this study
gem	gemF	AGGATACGTGTGTCGGGAATG	this study
	gemR	CGTTCGACCCGCAAATACAAA	this study
SMC1	SMC1F	AATGCCTTCGGTTCGCATAAG	this study
	SMC1R	TCAGACGCTAGAGATGGCAAG	this study
srp	srpF	CACCCCTTCTTCAACTTTCCG	this study
	srpR	CTAATGCCTGCCTTATCGCAC	this study
ush	ushF	TCTGCGATGTTATCTAAGCGCAGAGC	this study
	ushR	CGACTTCCTTCGCTCGCCTCG	this study
Atxn7	Atxn7F	GCAACGAATACCTGTGCGAC	this study

	Atxn7R	GGCAATTCGCGTCTCGTTAC	this study
bond	bondF	TGATGGCGTCGATCTTCTCG	this study
	bondR	TCTTGCGCAGCACAAAGAAC	this study
HetA	HetApromoterFW	ACCACGCCCAACCCCCAA	(2)
	HetApromoterRV	GCTGGTGGAGGTACGGAGACAG	(2)
Blood	Blood_5'_fw	TTATTACATGGCGACCGTGA	(2)
	Blood_5'_rv	CTCACACCTGTTGTCGCTGT	(2)
Burdock	Burdock_5'_fw	ATTAGAAGCGTCGGTCATCG	(2)
	Burdock_5'_rv	GGGCGCCAATTATCATTTTA	(2)
Jockey	Jockey_5'_fw	ATGGGAGATGAGCAATCGAG	(2)
	Jockey_5'_rv	GGGCAAAAACAACACAGCTT	(2)
Roo	Roo_5'_fw	CCTCTGCGTAGGCCATTTAC	(2)
	Roo_5'_rv	AAGGCTCGATTGACCAAATG	(2)
Gyspsy	Gypsy TSS F	ATTGCCGTTAAACATCATTGT	(3)
	Gypsy TSS R	GCTGAGGTTCGTCTTAGACAC	(3)

Supplementary Table S3. List of primer set sequences used for RT-qPCR

Target	Primer name	Sequence 5'-3'	Reference
α-Tubulin	TubF	ACACTTCCAATAAAAACTCAATATGC	(1)
	TubR	CCGTGCTCCAAGCAGTAGA	(1)
RP49	RP49F	TACAGGCCCAAGATCGTGAAG	(1)
	RP49R	GACGCACTCTGTTGTCGATACC	(1)
HeT-A	HeT-A_fwd	CGCGCGGAACCCATCTTCAGA	(3)
	HeT-A_rev	CGCCGCAGTCGTTTGGTGAGT	(3)
Blood	blood_fwd	AACAATAGAAAGAAGCCACCGAAC	(3)
	blood_rev	AGTCATGGACTATTGAGGGTGTTG	(3)
412	412F	GCGAAGGAATCTGGAGTCCC	(4)
	412R	TCGATGGTCGCTCACAAGTC	(4)
copia	copiaF	TCATGTGAAATTTGTGAACCCTG	(4)
	copiaR	AGGAAGTCTTGCCTGTTTACCATT	(4)
F-element	F-element F	GAGGTCAATCTGGTTGGTGGTT	(4)
	F-element R	TGATCACTCGCCTGTACTAATTCAC	(4)
Jockey	Jockey F	ACGACTCAATCTAGGGCTCGTG	(4)
	Jockey R	CGTCCATTCTCGTATTGATGG	(4)
Tirant	Tirant F	TCGGAAGAACCCAAATCAATATC	(4)
	Tirant R	GGCGGGCAGAATCGTTT	(4)
Mdg1	Mdg1 F	CGCCAGCAACAGCATTTG	(4)
	Mdg1 R	GCATACTCATTTCCGTTTTCTGATC	(4)
Idefix	Idefix F	CTACTACGATGGCGCACCTG	(4)
	Idefix R	TCCACTTGGTTGACGAATCCA	(4)
TART	TART F	GCCATCTCAAGGTCCTGCTACTAA	(4)

	TART R	TTCGATACCCGTTTCCTTTCA	(4)
gypsy	Gypsy F	GTTCATACCCTTGGTAGTAGC	(5)
	Gypsy R	CAACTTACGCATATGTGAGT	(5)
Burdock	Burdock-F	AGGGAAATATTTGGCCATCC	(5)
	Burdock-R	TTTTGGCCCTGTAAACCTTG	(5)
Gapdh2	GapdhF	CGTTCATGCCACCACCGCTA	this study
	GapdhR	CCACGTCCATCACGCCACAA	this study
dLsd1	dLsd1NtF	ACGGTTTTGTGTGGCCAAGA	(6)
	dLsd1NtR	GAGCGACTGATGTCCGGAAA	(6)
	dLsd1CtF	AACGCTAATCTCTTCGGACACG	(6)
	dLsd1CtR	ACTAGAGCGAGCAACACCGG	(6)
Ken	KenF	TCAAAGAAATCGGAGCAGCCT	this study
	KenR	GTCCTCCAGCAGGTTCCTTAT	this study
retn	retnF	AAAGAAGCCCGCCACTTTG	this study
	retnR	TGCTCTTCAAAGCTCCATCCA	this study
mid	midF	CGGACAGATCGTTTTGAACTCG	this study
	midR	GCAATTCCTTGGGATTCGACG	this study
lola	lolaF	GCGGTTTGCTGGCTAAATTCT	this study
	lolaR	TGTGTCAGCAGTTATGTGTTGTG	this study
Src64B	Src64BF	GACCTTCAGAGCACAATCCCA	this study
	Src64BR	AGAGCGTTTTTGCTGTATGCC	this study
CHES1-like	CHES1-likeF	GAGCCCTTCCAGAATGGTAGT	this study
	CHES1-likeR	CCAGCTCCTCCCTGGTTATTC	this study
Sxl	SxlF	AGTTCCACTCGTGACAAGTCC	this study
	SxlR	CCCACCACTCGCCATCTTA	this study
rRNA	rRNAF	CTTAGTAGCGGCGAGCAAAC	this study
	rRNAR	TCTATGGGTAAATGGCCTCA	this study
dGATAd	dGATAdF	GTCAAATTCTTTGCCGGCGT	this study
	dGATAdR	TTCTCTCTTACGCGACCTCA	this study
serpent	srpF	TTTCTGTGAAAATCGCGCCG	this study
	srpR	GATCCCGACCCTATAACCGC	this study
ush	ushF	GTGCGTAAATGTTTCGGCGT	this study
	ushR	CTCGGTTTTTCATCGCTCCG	this study
pnt	pntF	AACAGATCCTGATGAGGTGGC	this study
	pntR	GCCGTCTTGTGGATGATGTTT	this study
SMC1	SMC1F	AATGGAGAGAGCGTATCGAGC	this study
	SMC1R	ATGTTCTCAACAGCTCCCTGG	this study
piwi	piwiF	AGGCACTTCAGAGACCAAAGG	this study
	piwiR	TATGAATTCCTGGGGGCTTCG	this study

Supplementary Table S4. List of primer set sequences used for dsRNA in S2

Target	Primer name	Sequence 5'-3'	Ref
dLsd1	RNAidLsd66_F	CTAATACGACTCACTATAGGGAGAATTAGCGAC GATTCCGATGG	(6)
	RNAidLsd816_ R	CTAATACGACTCACTATAGGGAGTGCCCCAATC ACTATGACTTTG	(6)
serpent	RNAidSRP_BK N32327_F	TAATACGACTCACTATAGGGTATGGTCACCTCA ACACCGA	this study
	RNAidSRP_BK N32327_R	TAATACGACTCACTATAGGGAGTAGGGTGGGG AAGGTCTC	this study
dGATAd	RNAidGatad_ BKN26148 _F	TAATACGACTCACTATAGGGGATAACCCGCTAT CAGCCAA	this study
	RNAidGatad_ BKN26148 _R	TAATACGACTCACTATAGGGTTGCCGTAGTTAT CGGTTCC	this study

Supplementary Table S5. List of motifs enriched at dLsd1 peaks

Motif sequence	TF	P-value (adj. fisher)
CATCGATAACT	GATA factor	3.12E-240
TACCTCATATCGATA	GATA factor, Beaf-32	2.56E-176
TTGCTCTCTC	trl	8.91E-56
TCTAATTA	inv	8.15E-14
TAACA	-	4.41E-09
GTGACGTCGCCGCGA	Mad	3.64E-07
TTCGTG	-	6.38E-07
AAACA	-	1.39E-05
CTGGCGCT	brk	1.41E-05
AGTAACAGA	ovo	0.00632929
AGTAACAGA	prd	0.006329295
TATCTAGGTGGAGCT	CTCF	0.01127397
TAGTAAACTTA	Br(var.4)	0.023718933

Supplementary Table S6. dGATA factors expression levels in ovaries and S2 cells according to modENCODE RNA-Seq and FlyAtlas MicroArray analysis

Transcript	Fly Base ID	FlyAtlas (ovary)	modENCODE mRNASeq (ovary mated)	modENCODE mRNASeq (S2R+)
**Pannier/dGATAa**	FBgn0003117	0.8 (no expression)	0 (no expression)	0 (no expression)
**Serpent/dGATAb**	FBgn0003507	201.9 (moderate expression)	39 (moderately high expression)	84 (high expression)
**Grain/dGATAc**	FBgn0001138	0.5 (no expression)	0 (no expression)	0 (no expression)
**dGATAd**	FBgn0032223	251 (moderate expression)	28 (moderate expression)	11 (moderate expression)
**dGATAe**	FBgn0038391	2.5 (no expression)	0 (no expression)	1 (very low expression)

Supplementary Table S7. List of the differentially expressed transcripts in ovaries depleted for dLsd1 by RNAi compared to control ovaries (xlsx)

Supplementary Table S8. List of dLsd1 direct targets (xlsx)

Supplementary Table S9. Fold changes and False Discovery Rates (FDR) of repeats for dLsd1 samples against input and pre-immune (xlsx)

SUPPLEMENTARY REFERENCES

1. Miles, W.O., Lepesant, J.M., Bourdeaux, J., Texier, M., Kerenyi, M.A., Nakakido, M., Hamamoto, R., Orkin, S.H., Dyson, N.J. and Di Stefano, L. (2015) The LSD1 Family of Histone Demethylases and the Pumilio Posttranscriptional Repressor Function in a Complex Regulatory Feedback Loop. *Molecular and cellular biology*, **35**, 4199-4211.
2. Wang, S.H. and Elgin, S.C. (2011) Drosophila Piwi functions downstream of piRNA production mediating a chromatin-based transposon silencing mechanism in female germ line. *Proceedings of the National Academy of Sciences of the United States of America*, **108**, 21164-21169.
3. Donertas, D., Sienski, G. and Brennecke, J. (2013) Drosophila Gtsf1 is an essential component of the Piwi-mediated transcriptional silencing complex. *Genes & development*, **27**, 1693-1705.

4. Basquin, D., Spierer, A., Begeot, F., Koryakov, D.E., Todeschini, A.L., Ronsseray, S., Vieira, C., Spierer, P. and Delattre, M. (2014) The Drosophila Su(var)3-7 gene is required for oogenesis and female fertility, genetically interacts with piwi and aubergine, but impacts only weakly transposon silencing. *PloS one*, **9**, e96802.
5. Chen, Y.C., Stuwe, E., Luo, Y., Ninova, M., Le Thomas, A., Rozhavskaya, E., Li, S., Vempati, S., Laver, J.D., Patel, D.J. *et al.* (2016) Cutoff Suppresses RNA Polymerase II Termination to Ensure Expression of piRNA Precursors. *Molecular cell*, **63**, 97-109.
6. Di Stefano, L., Walker, J.A., Burgio, G., Corona, D.F., Mulligan, P., Naar, A.M. and Dyson, N.J. (2011) Functional antagonism between histone H3K4 demethylases in vivo. *Genes & development*, **25**, 17-28.

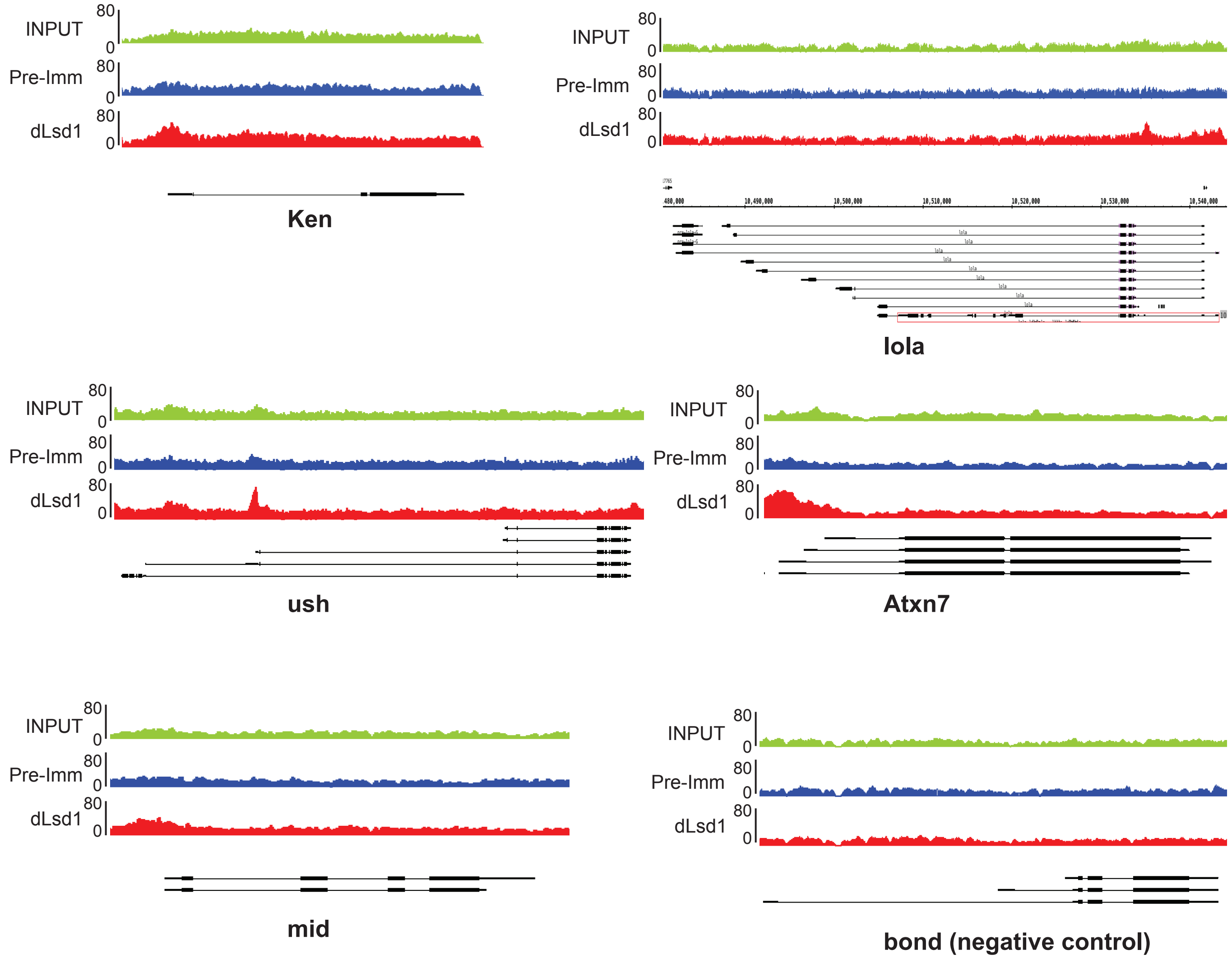

Supplementary Figure S1

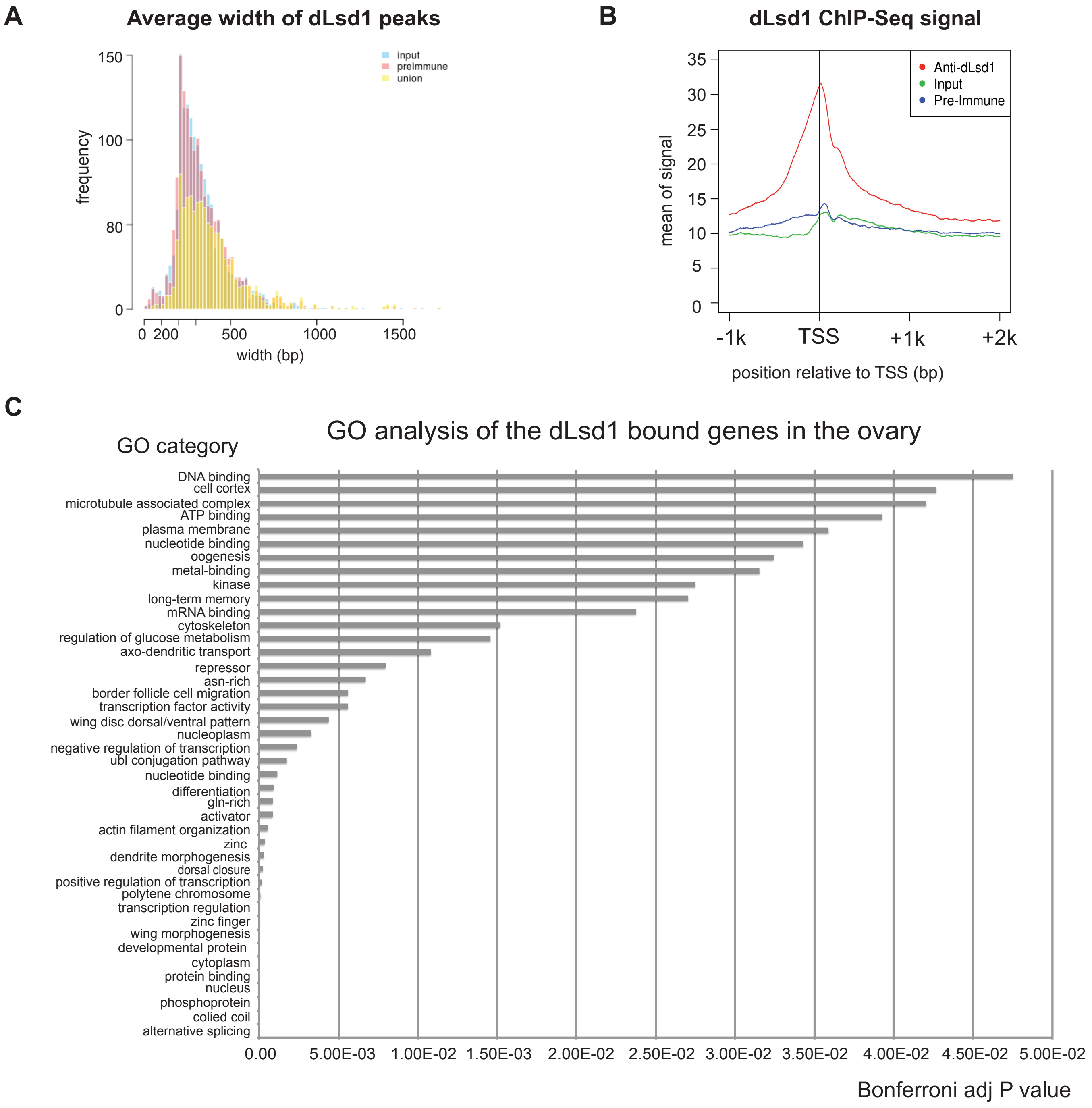

Supplementary Figure S2

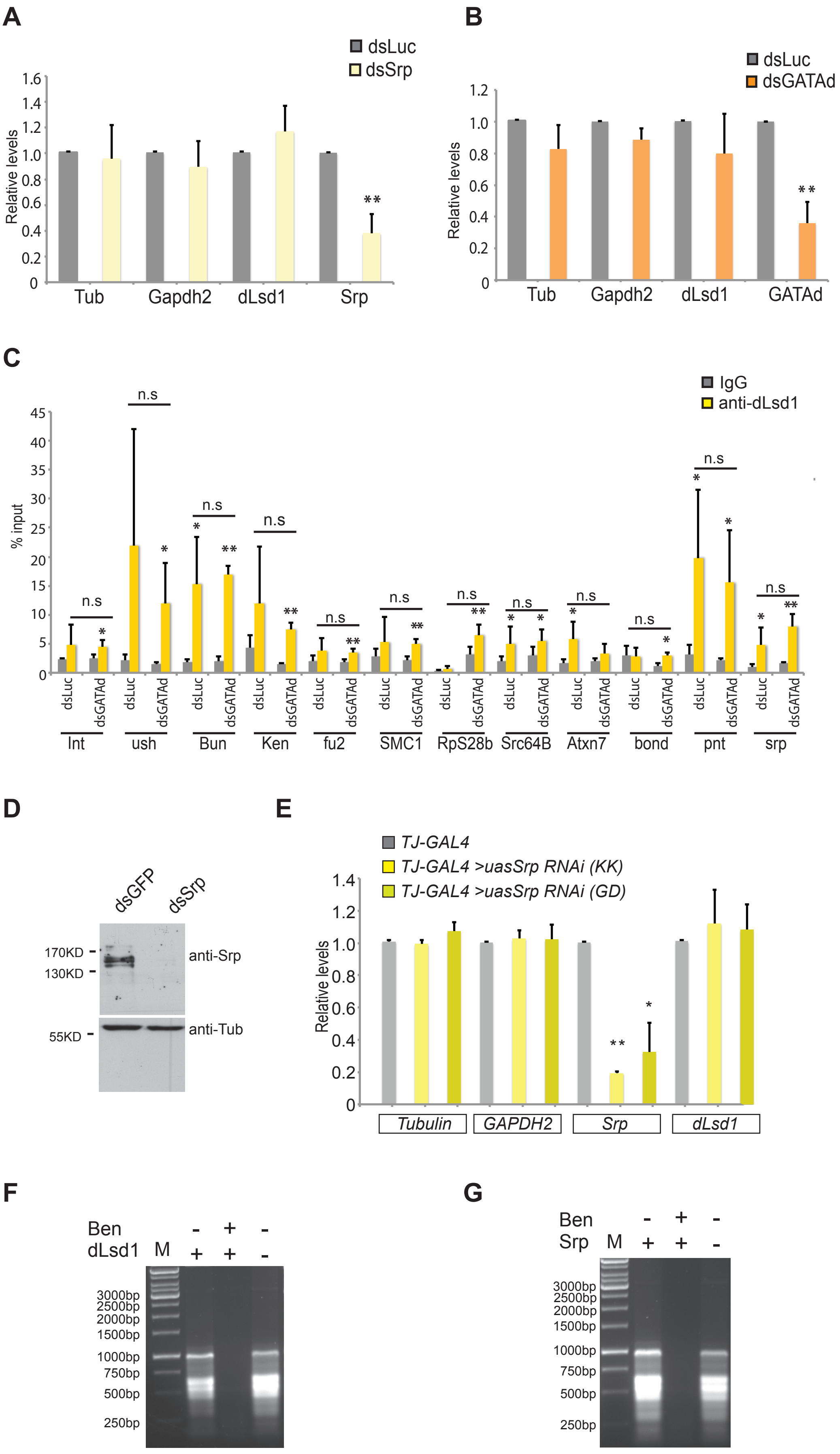

Supplementary Figure S3

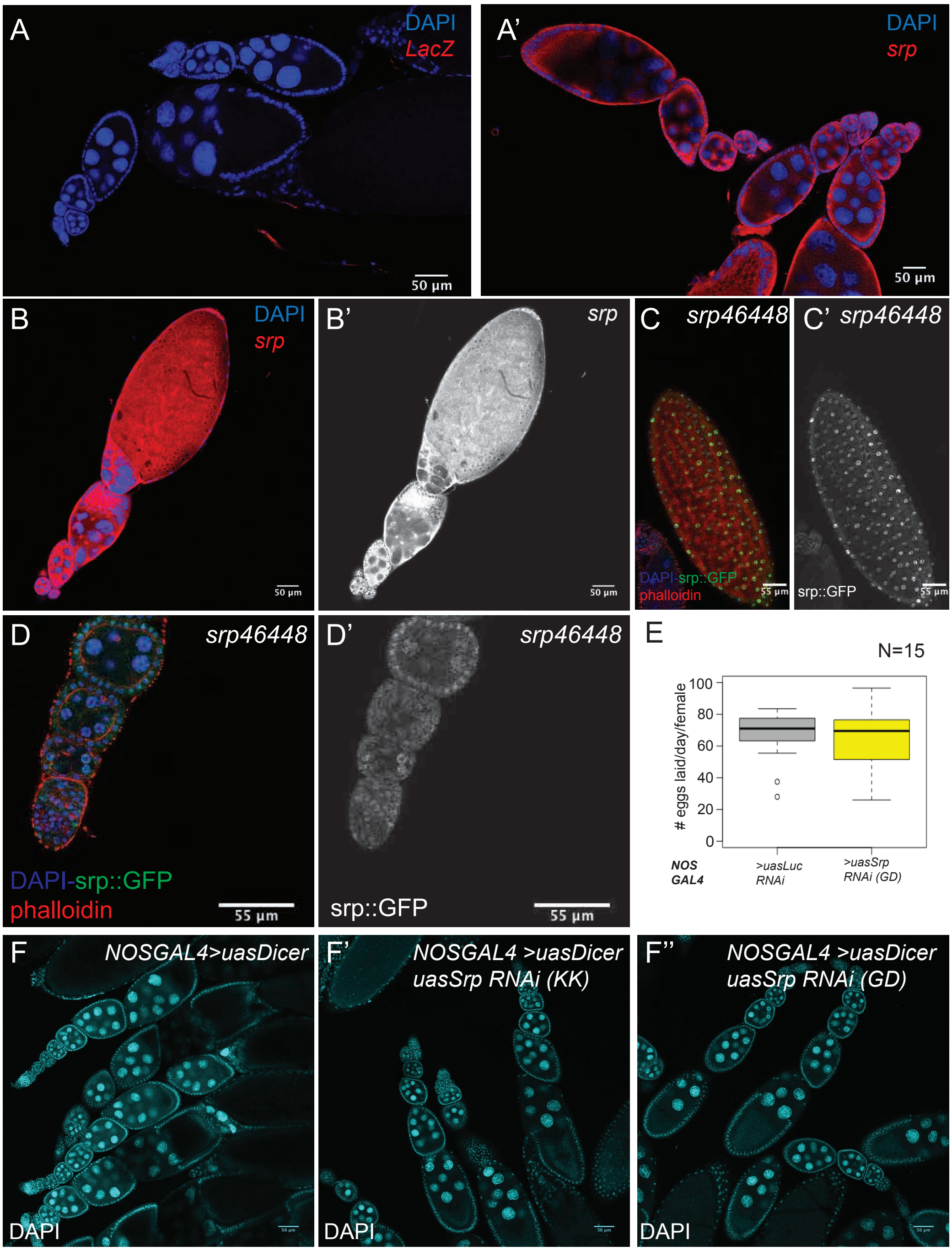

Supplementary Figure S4

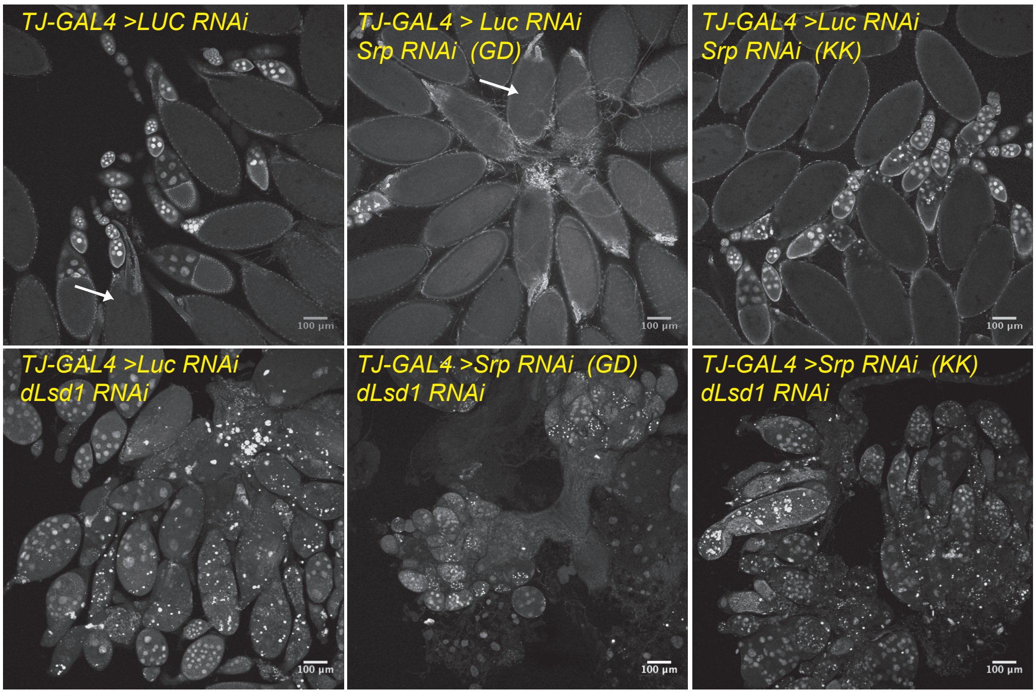

Supplementary Figure S5

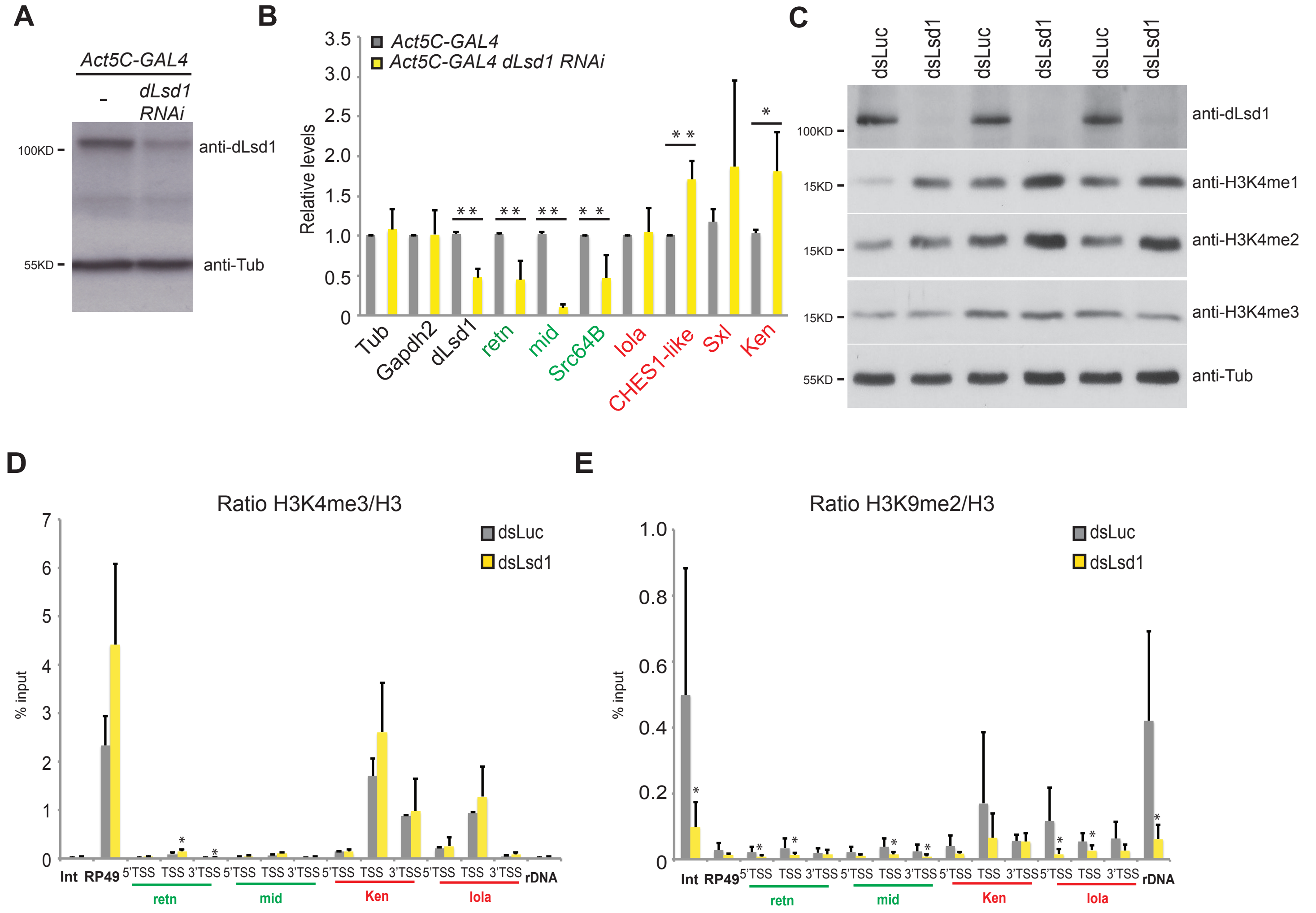

Supplementary Figure S6

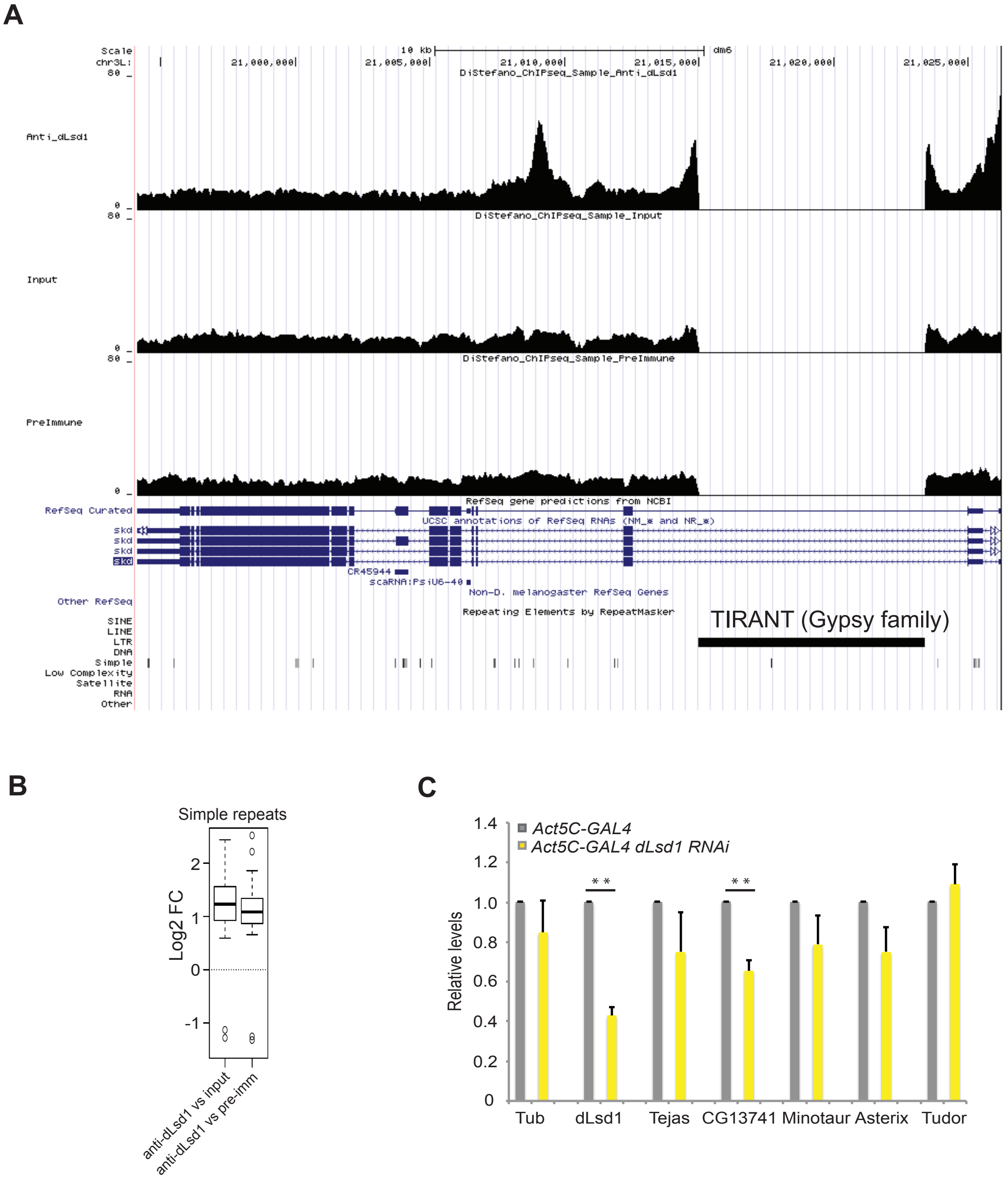

Supplementary Figure S7

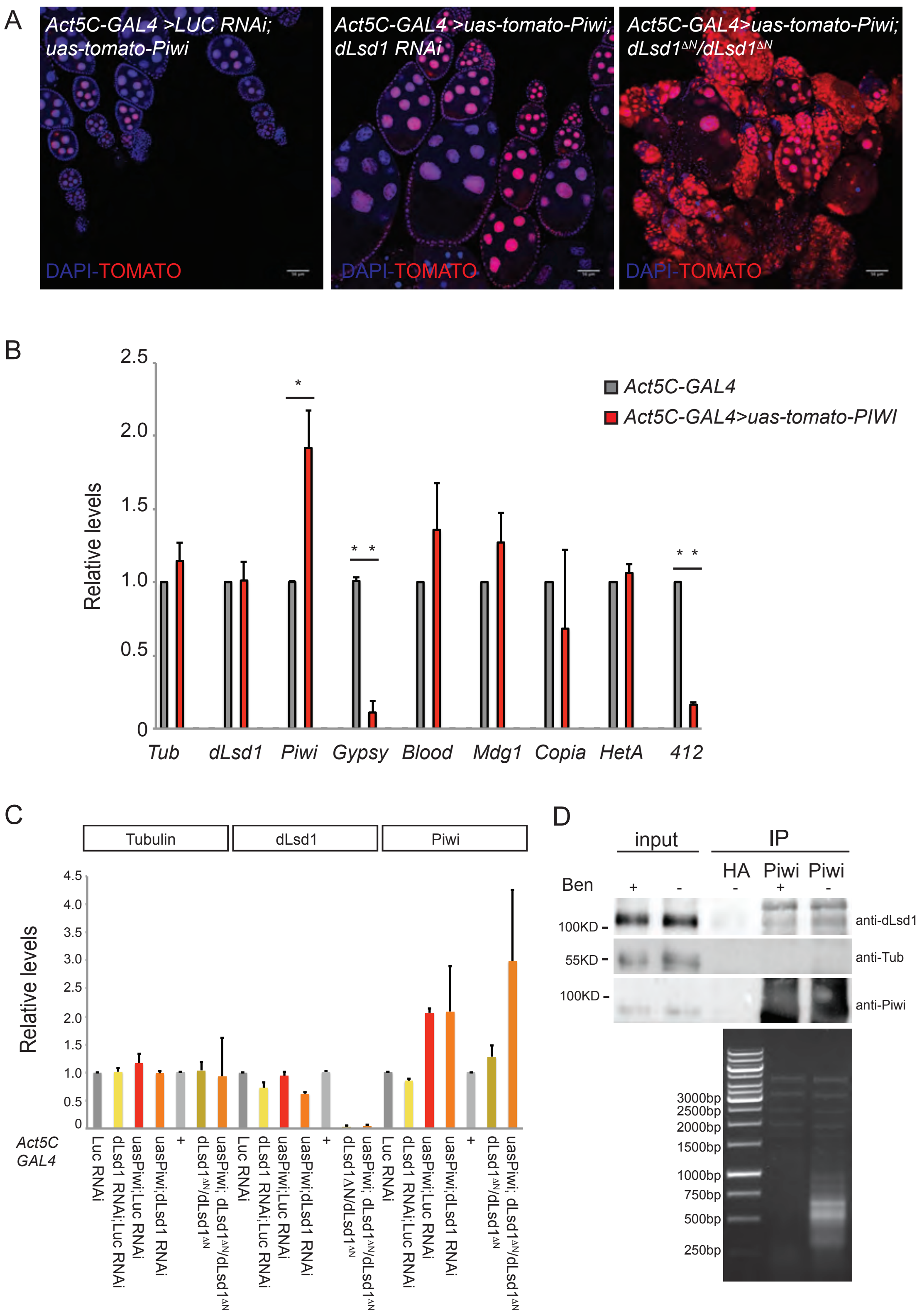

Supplementary Figure S8
